# Dorsal Bed Nucleus of the Stria Terminalis-Subcortical Output Circuits Encode Positive Bias in Pavlovian Fear and Reward

**DOI:** 10.3389/fncir.2021.772512

**Published:** 2021-12-14

**Authors:** Nadia Kaouane, Sibel Ada, Marlene Hausleitner, Wulf Haubensak

**Affiliations:** ^1^Research Institute of Molecular Pathology (IMP), Vienna Biocenter (VBC), Vienna, Austria; ^2^Department of Neuronal Cell Biology, Center for Brain Research, Medical University of Vienna, Vienna, Austria

**Keywords:** BNST, fear, reward, Pavlovian conditioning, PVH, PAG

## Abstract

Opposite emotions like fear and reward states often utilize the same brain regions. The bed nucleus of the stria terminalis (BNST) comprises one hub for processing fear and reward processes. However, it remains unknown how dorsal BNST (dBNST) circuits process these antagonistic behaviors. Here, we exploited a combined Pavlovian fear and reward conditioning task that exposed mice to conditioned tone stimuli (CS)s, either paired with sucrose delivery or footshock unconditioned stimuli (US). Pharmacological inactivation identified the dorsal BNST as a crucial element for both fear and reward behavior. Deep brain calcium imaging revealed opposite roles of two distinct dBNST neuronal output pathways to the periaqueductal gray (PAG) or paraventricular hypothalamus (PVH). dBNST neural activity profiles differentially process valence and Pavlovian behavior components: dBNST-PAG neurons encode fear CS, whereas dBNST-PVH neurons encode reward responding. Optogenetic activation of BNST-PVH neurons increased reward seeking, whereas dBNST-PAG neurons attenuated freezing. Thus, dBNST-PVH or dBNST-PAG circuitry encodes oppositely valenced fear and reward states, while simultaneously triggering an overall positive affective response bias (increased reward seeking while reducing fear responses). We speculate that this mechanism amplifies reward responding and suppresses fear responses linked to BNST dysfunction in stress and addictive behaviors.

## Introduction

Responding to environmental stimuli that predict positive and negative outcomes with appropriate affective behaviors is critical to survival. Fearful stimuli elicit defensive coping strategies (e.g., freezing), while rewarding stimuli trigger reward-seeking behaviors. Typically, research delineates the neuronal processing of one affective state at a time, i.e., fear (LeDoux, 2000; Haubensak et al., 2010; Tovote et al., 2015) and reward-related states (Everitt et al., 1999). Interestingly, various neural processing hubs contribute to both fear and reward, like the amygdala (Paton et al., 2006; Shabel and Janak, 2009; Beyeler et al., 2016; Kim et al., 2016), and the ventral tegmental area (VTA) (Cohen et al., 2012; Lammel et al., 2012). We questioned how a single network node differentiates oppositely valenced affective states to drive appropriate behavioral responses (Calder et al., 2001; Lammel et al., 2014; Namburi et al., 2016).

The bed nucleus of the stria terminalis (BNST) constitutes a key relay and integration point (Lebow and Chen, 2016; Ch'ng et al., 2018) between subcortical limbic structures (e.g., amygdala, hippocampus; Dong et al., 2001; Dong and Swanson, 2004) and downstream regions [e.g., periaqueductal gray (PAG), paraventricular hypothalamus (PVH; Cullinan et al., 1993; Dong and Swanson, 2006)] that underlies appropriate behavioral responses to emotional stimuli (Herman et al., 2003; Johnson et al., 2016). Although the BNST plays a critical role in anxiety-like or long-lasting sustained fear behaviors (Walker and Davis, 1997; Jennings et al., 2013a; Kim et al., 2013), evidence now indicates a direct role of the BNST in phasic fear states (Duvarci et al., 2009; Haufler et al., 2013; De Bundel et al., 2016; Bjorni et al., 2020). Yet, other evidence shows the BNST, mostly its dorsal part (dBNST), can modulate both aversive and rewarding phenotypes (Jennings et al., 2013a; Kim et al., 2013; Giardino et al., 2018; Girven et al., 2020). Based on these data, we hypothesized that the dBNST is a critical brain region for processing both fear and reward-related states and behaviors.

The BNST is a heterogeneous structure, comprised of several subdivisions and projection targets, suggesting a complex functional modular organization. However, how these functional modules in the BNST differentially process opposing affective states remains unclear. In humans, this dichotomy exists in psychiatric disorders like anxiety disorders and addiction, which have contradictory comorbidities (Kessler et al., 1995). These opposing features have been linked to dysregulated fear and reward processing in the BNST (Avery et al., 2016; Lebow and Chen, 2016; Ch'ng et al., 2018). Such BNST dysregulation may, in fact, underly increased reward-seeking in rodent models of drug abuse (Erb et al., 2001; Shaham et al., 2003), as increased BNST anxiety may evolve into drug-seeking. Using a combined Pavlovian reward and fear conditioning paradigm (Shabel and Janak, 2009; Kargl et al., 2020) and circuit physiology, we examined how dBNST and its key outputs to PVH and PAG differentially encode and control fear and reward stimuli and behaviors.

## Materials and Methods

### Experimental Subjects

All experiments were conducted using 2 to 6-month-old male mice (C57Bl/6J, Charles River, Germany). They were group housed by 2–5 and in a temperature- and humidity-controlled room under a 14-h light/10-h dark cycle, an alternative cycle commonly used in rodent studies. Before the experiments, they had *ad libitum* access to food and water and handled for several days. The experiments took place during the light phase. All animal care and behavioral tests were conducted in agreement with the Austrian (BGBl nr. 501/1988, idF BGBl I No. 162/2005) and European (Directive 86/609/EEC of 24 November 1986, European Community) legislation on animal experimentation and covered by the license M58/002220/2011/9.

### Stereotactic Surgery

All mice were between 10 and 12 weeks for surgery. Mice were deeply anesthetized with isoflurane (IsoFlo^®^, Abbot Laboratories, North Chicago, IL, USA; induction, 2.3%; maintenance, 1.5–2%; airflow, 180 ml/min) and placed in a stereotactic frame (David Kopf Instruments, Tujunga, CA, USA). Gentamicin ointment (Refobacin^®^ 3 mg/g, Merck, Darmstadt, Germany) was used to protect the animals' eyes, and their body temperature was maintained at 36°C with a heating pad controlled by a rectal thermometer (DC temperature controller FHC, Bowdoin, ME, USA). After injecting 0.1 ml of Lidocaine (Xylanaest 1%, Gebro Pharma, Fieberbrunn, Austria) under the skin as analgesia, the skull was exposed and perforated with a stereotactic drill at the desired coordinates relative to Bregma (Franklin and Paxinos, 2007). For post-operative care, mice were supplied with 250-mg/l Carprofen (Rimadyl, Pfizer, New York, NY, USA) and 300-mg/l Enrofloxacine (Baytril, KVP pharma, Kiel, Germany) in drinking water for 7 days.

For the inactivation experiment, two guide cannulas (5-mm length, 24 GA, C316GS-4/SPC, Plastics One, Roanoke, VA, USA) were implanted bilaterally 1 mm above the dBNST (AP + 0.26, ML ± 1.8, DV – 3.4) under a 15° angle toward the midline in the coronal plane to avoid damaging the lateral ventricle. All implants were fixed to the skull with dental cement (SuperBond C&B kit, Prestige Dental Products, Bradford, UK).

For optogenetic experiments, mice were injected bilaterally into the dBNST (AP + 0.26, ML ± 0.9, DV – 4.) with GFP (AAV5.hsyn.eGFP.WPRE.hGH, Penn Vector Core, Philadelphia, PA, USA, titer 1.15E + 13 GC/ml, 10–20 nl) or ChR2 [AAV2/5.hsyn.hChR2 (H134R).eYFP.WPRE, Penn Vector Core, titer 1.30 E + 13 GC/ml, 10–20 nl]. A Micro4 Micro Syringe Pump controller (World Precision Instruments, Sarasota, FL, USA) was used to regulate injection volumes with a rate of 5–10 nl/min. After the injection was completed, the glass needle was left in place for supplemental 5–10 min to guarantee complete injection and diffusion of the virus. The mice were then implanted with optic fiber(s) above either PVH or the l/vl PAG. One optic fiber (Doric lenses, Quebec, Canada, 400 μm, 0.53 NA) per mouse was implanted 0.5 mm above the PVH (AP – 0.5, ML 0, DV – 4.5). Placing a single fiber at the midline ensured bilateral illumination of PVH neurons, which accumulated close to the midline on both sides of the 3rd ventricle. Two optic fibers (Doric lenses, 200 μm, 0.53 NA) per mouse were implanted 0.5 mm above the l/vl PAG (AP – 4.75, ML ± 0.98, DV – 2.25) under a 10° angle toward the midline in the coronal plane to allow enough space between the two fibers, which were implanted relatively close to the midline.

For the calcium imaging experiments, mice were injected unilaterally into the left or right dBNST with an AAV carrying a Ca^2+^ indicator (AAV1.Syn.GCaMP6f.WPRE.SV40, Penn Vector Core, CS1107, titer 4.27E + 12 GC/ml, 80 nl, 20 nl/min). For calcium recording in dBNST-projecting neurons to PVH or PAG, another cohort of mice was injected unilaterally with a retrograde canine-adenovirus expressing Cre-recombinase into the PVH or the l/vlPAG (CAV2.Cre, Montpellier Vector Platform, France, titer 5.50E + 12 GC/ml, dilution 1:6 in PBS, 60 nl, 10 nl/min) and with an AAV carrying a Cre-dependent Ca^2+^ indicator into the dBNST (AAV1.hsyn.DIO.GCaMP6f.WPRE, Penn Vector Core, AV-1-PV2819, 1.00 E + 13 GC/ml, 80 nl, 20 nl/min). To ensure localization and visualization of the CAV2.Cre injection during histology, CTB-Alexa Fluor 555 (Invitrogen, Carlsbad, CA, USA) was also injected with the virus (one-part CTB for one-part CAV2.Cre). At least 4 weeks after injection, a microendoscope (Inscopix, Palo Alto, CA, USA, lens probe Part ID: 1050-002182, 0.5-mm diameter, 6.1-mm length) was implanted above the dBNST. After a 1-week recovery period, a baseplate was cemented onto the skull (Inscopix microscope baseplate, Part ID: 1050-002192) and covered (Inscopix microscope objective lens cover, Part ID: 1050-002194).

### Combined Pavlovian Reward and Fear Conditioning

The combined Pavlovian reward and fear conditioning paradigm (Kargl et al., 2020) occurred in four identical experimental chambers (16.5-cm wide × 16.5-cm deep × 30.5-cm high, H10-11M-TC, Coulbourn Instruments, Allentown, PA, USA) encased in a sound-attenuating shell. Above the chamber, a custom-made house light provided illumination (around 10 lux), an infrared spotlight (Kemo Electronic, Geestland, Germany) improved mice detection, a speaker (Audiocomm, Vienna, Austria) provided sounds designed using Audacity software (http://www.audacityteam.org) with a maximal sampling frequency of 192 kHz and played from a Terratec sound card (Alsdorf, Germany), and a video camera (Basler, Ahrensburg, Germany) monitored the animal's behavior. Port training, reward conditioning, and test sessions utilized the original experiment chambers combined with a grid floor (H10-11M-TC-NSF, Coulbourn Instruments), cleaned with 70% ethanol before each mouse, with the house light turned on and with access to a port located in the center of the right wall. In the port, delivery of the solution was controlled by opening an electronic valve (003-0096-900, Parker Hannifin, Cleveland, OH, USA) from a syringe located outside the chamber until a drink tube in the port. Photocell sensors mounted at the entry of the port (RS components, Corby, UK) and on either side of the drink tube (H24-01 M, Coulbourn Instruments) measured port visits and licking/drinking via beam breaks of the animal's head and tongue, respectively. Fear conditioning occurred in a custom-made triangular cartridge inserted to the experiment chambers, combined with a stainless-steel shock floor (H10-11M-TC-SF, Coulbourn Instruments), cleaned with lemon-scented diluted cleaning solution before each mouse, with the house light turned off and no access to the port. The day before the start of the experiments, body weight was measured, and water deprivation prompted mice to seek liquid reward. Throughout the duration of the training, mice were weighed before each session and were given water after training to maintain 82–90% of their free drinking weight. The mice were trained, and then during a 50-min port training session to collect a drop of sucrose (~5 μL, 5% wt/vol) randomly delivered 36 times on a 100 ± 30 s interstimulus interval. Only after successful port training, the mice underwent eight sessions of reward conditioning where, after a baseline of 1 min, reward-CS presentation (white noise, 50-ms pips for 10 s at 0.9 Hz, 75 dB) was immediately followed by sucrose delivery. Each session lasted 50 min and consisted of 24 trials with a randomized intertrial interval (ITI) of 110 ± 20 s. The day after the last session of reward conditioning, the mice were submitted to a fear-conditioning session, where after a baseline of 2 min, fear-CS presentation (3 kHz tone, 2-s pips for 10 s at 0.4 Hz, 75 dB) was immediately followed by a footshock (1 s, 0.5 mA) delivered to the floor via an external shocker (H13–15, Coulbourn Instruments). The session lasted 10.5 min and consisted of 5 trials with a randomized ITI of 100 ± 30 s. The next day(s), the mice were tested for the expression of the correct behavioral response during the presentation of fear-CS and reward-CS in the absence of shock or sucrose delivery. After a baseline of 1 min, the mice received 4 presentations of fear-CS and reward-CS with a randomized ITI of 60 ± 10 s within the same session. For the inactivation experiment, the test first consisted of 4 presentations of reward-CS, and then four presentations of fear-CS. Reward-CS were presented before the fear-CS to avoid any fearful states during the reward-CS trials. For optogenetic experiments, reward-CS and fear-CS presentations were associated with laser illumination during the test. One week later, a second test was repeated after retraining to reward conditioning to confirm the results of the first test (results during reward-CS trials showed in Figure 5 represent the average of the two tests). Due to a probable ceiling effect when using a high footshock intensity, the mice were retrained 2 weeks later to fear conditioning with a lower footshock intensity (0.1 mA) and tested the day after with fear-CS presentations, coupled with a laser. For calcium experiment, reward-CS and fear-CS were presented pseudo-randomly. The experimental procedures and recordings of visits and licks were performed with custom MATLAB programs (R2015b, MathWorks, Natick, MA, USA). Reward-seeking behavior was scored as the rate of port visits during the CSs compared to a baseline period (10-s periods before the CSs). Freezing behavior, defined as a lack of all movement, except respiratory-related movements (Fanselow, 1980) was scored on recorded videos (20 frames/s) with Ethovision XT 12 (Noldus Information Technology, Wageningen, the Netherlands) offline (0.5-s minimum time immobile, <2.5% pixel change).

### BNST Inactivation

The mice were handled and habituated to intracerebral injection procedures for several days prior to the behavioral experiment. On the day of testing, the animals were brought to the infusion room and received intra-dBNST bilateral infusions (100 nl per side) of artificial cerebrospinal fluid (aCSF, Harvard Apparatus, Holliston, MA, USA) or the GABA_A_ receptor agonist muscimol (1 mg/ml, M1523, Sigma, St Louis, MO, USA). Infusions were performed using internal cannulas (6-mm length, 31GA, C316IS/SPC, Plastics One) inserted into the guide cannulas. The internal cannulas were attached with polyethylene catheter tubing to 1-μl Hamilton syringes (Reno, NV, USA), which were controlled by an infusion pump (Pump 11, Elite, Harvard Apparatus). The syringes were fixed in a constant rate infusion pump (100 nl/min). The cannulas were left in place for an additional 3 min before removing them to guarantee drug diffusion away from the injection site. Behavioral testing started 15 min after infusion.

### CTB Tracing

Dorsal BNST neurons projecting to the PVH and PAG were retrogradely labeled using cholera toxin subunit B (CTB) conjugated with a different fluorophore. All the mice were between 10 and 12 weeks at the time of surgery, following previously described procedures. Each mouse received bilateral CTB injections into the PVH and l/vlPAG. To label the projections, 10 nl (per injection) CTB-Alexa Fluor 488 (Invitrogen) was injected into the PVH, and 10 nl (per injection) CTB-Alexa Fluor 647 (Invitrogen) was injected into the l/vlPAG. For both regions, an injection speed of 5 nl/min was used. One week later, the mice were deeply anesthetized with a mixture of 10 mg/ml ketamine (Ketasol, OGRIS Pharma, Wels, Austria) and 0.8 mg/ml medetomidine hydrochloride (Domitor, ORION Pharma, Espoo, Finland) in 1 × PBS. They were then intracardially perfused with heparin solution (Sigma-Aldrich, 10 U/ml Heparin/PBS), followed by cold 4% PFA. Brains were immediately removed, post-fixed overnight in 4% PFA at 4°C, and transferred to 1 × PBS. The brains were cut into 60-μm thick coronal slices at a vibratome. Sections were rinsed in 1 × PBST (0.1% Triton X-100 in 1 × PBS), incubated for 2 h at room temperature with DAPI (Invitrogen D3571, 1:1,000) and mounted onto microscopy slides with Fluorescence Mounting Medium (Dako, S302380, Vienna, Austria). Images of CTB labeling in the dBNST were acquired with a confocal microscope (LSM 700, Zeiss, Göttingen, Germany). A region of interest (ROI) was defined and cropped following the anatomical landmarks of the dBNST (Ju and Swanson, 1989; Franklin and Paxinos, 2007). Labeled and colocalized CTB cells were counted manually in this ROI using FIJI (ImageJ software, National Institutes of Health, USA). DAPI-positive nuclei were counted using Definiens Developer XD software (Definiens, Carlsbad, CA, USA). Images of CTB injections in PVH and l/vlPAG were acquired using a Mirax slide scanner (Zeiss).

### Optogenetic Manipulation

The mice were handled and habituated to attach the optic fiber cables (Doric lenses, Quebec, Canada) to the fiber implants for several days prior to behavioral experiments. ChR2 activation was performed with 473 nm or a 457-nm laser, delivering 5-ms pulses with a frequency of 20 Hz, at an intensity of 8–10 mW at the fiber tip, measured with a power meter (PM100D, Thorlabs, Newton, NJ, USA). The laser was triggered by custom MATLAB scripts (R2015b, MathWorks) during test sessions throughout CSs presentations only. From a total of 37 mice, 6 animals did not complete the behavioral task because of sickness developed after surgery or loss of their optic fiber(s). Moreover, 5 animals were excluded from the analysis due to incorrect viral expression and/or fiber(s) placement.

### Calcium Imaging

Several days prior to the experiments, the mice were habituated to the microscope-mounting process using a dummy microscope. On recording days, the microscope (Inscopix) was attached to the baseplate before the start of the behavioral experiment. Acquisition of Ca^2+^ signals was conducted using the nVista HD System v2.0.32 (*In vivo* Rodent Brain Imaging System, Inscopix) at 20 fps. During each session of the behavioral protocol and for each CS trial, Ca^2+^ recording started 15 s before the CS onset and finished 15 s after the CS offset. Data were analyzed with Mosaic v1.2.0 software (Inscopix). The videos were concatenated per session, down-sampled 2 × 2 (time × space), motion corrected, and the Ca^2+^ signal was calculated as the relative change of fluorescence over the entire recording session [ΔF/F_0_ = (F_t_-F_0_)/F_0_]. The individual neurons and their Ca^2+^ traces were extracted by applying PCA-ICA analysis. Spatial filters obtained by PCA-ICA were manually selected to avoid duplicates or false units in further analysis. Ca^2+^ traces were then low-pass filtered at 0.5 Hz, and Ca^2+^ events were automatically detected with an event threshold >5 s.d. and τ_off_ >0.5 s. Exported events were further analyzed with Neuroexplorer software v5.114 (Plexon, Dallas, TX, USA). Neuronal events were exported as peri-event time histogram (PETH, 0.5-s bin) and z-scored per recording session. Only data within −8 to 18 s relative to the CS onset were considered and were binned at 1 s. Average z-scores were aligned with CS or the behavior onset (freezing epochs were filtered at 1 s; visits and licks were filtered at 5 s). Responders dBNST cells were defined as showing a positive response (trial-averaged neuronal responses above a z-score of 1.65) within the first 4 s (1-s bin) of the fear-CS, reward-CS, or the behavior onset.

### Histological Analysis

All the mice in the inactivation experiment received, after completion of the behavioral procedure, intra-dBNST bilateral infusions (100 nl per side) of muscimol conjugated with a fluorophore (BODIPY-TMR-X muscimol conjugate, M23400, Invitrogen, 5 mM in aCSF). The mice were rapidly decapitated 45 min later, and their brains were immediately placed in 4% paraformaldehyde (PFA) in 1 × PBS (pH 7.4) for fixation at 4°C overnight. The brains were then stored in 1 × PBS and cut on a vibratome into 60-μm coronal sections. The sections were mounted on slides and observed at Zeiss fluorescence stereomicroscope for verifying the location of the Muscimol-BODIPY intracerebral infusion.

The mice in the optogenetic and calcium experiments were killed after completing the behavioral procedure, and their brains were processed for histological analysis as described above. After vibratome sectioning, the sections were counterstained with DAPI and mounted on slides. Images were acquired using a confocal microscope and a Mirax slide scanner.

### Statistics

Statistical analyses were performed using GraphPad Prism software (Version 8, La Jolla, CA, USA). ANOVAs were used with 1 or 2 dependent factors, repeated or non-repeated, followed by *post-hoc* analyses as needed (a two-stage step-up method of Benjamini, Krieger, and Yekutieli or Fisher's LSD test). The chi-squared test was used to compare cell numbers among the CTB-projecting neurons. Two-tailed paired or unpaired *t*-tests were used to evaluate the statistical differences between two groups. Differences between groups were considered statistically significant at a value of *p* < 0.05.

## Results

### dBNST Neurons Are Necessary for the Expression of Fear and Reward-Related Behaviors

**Figure 1:**
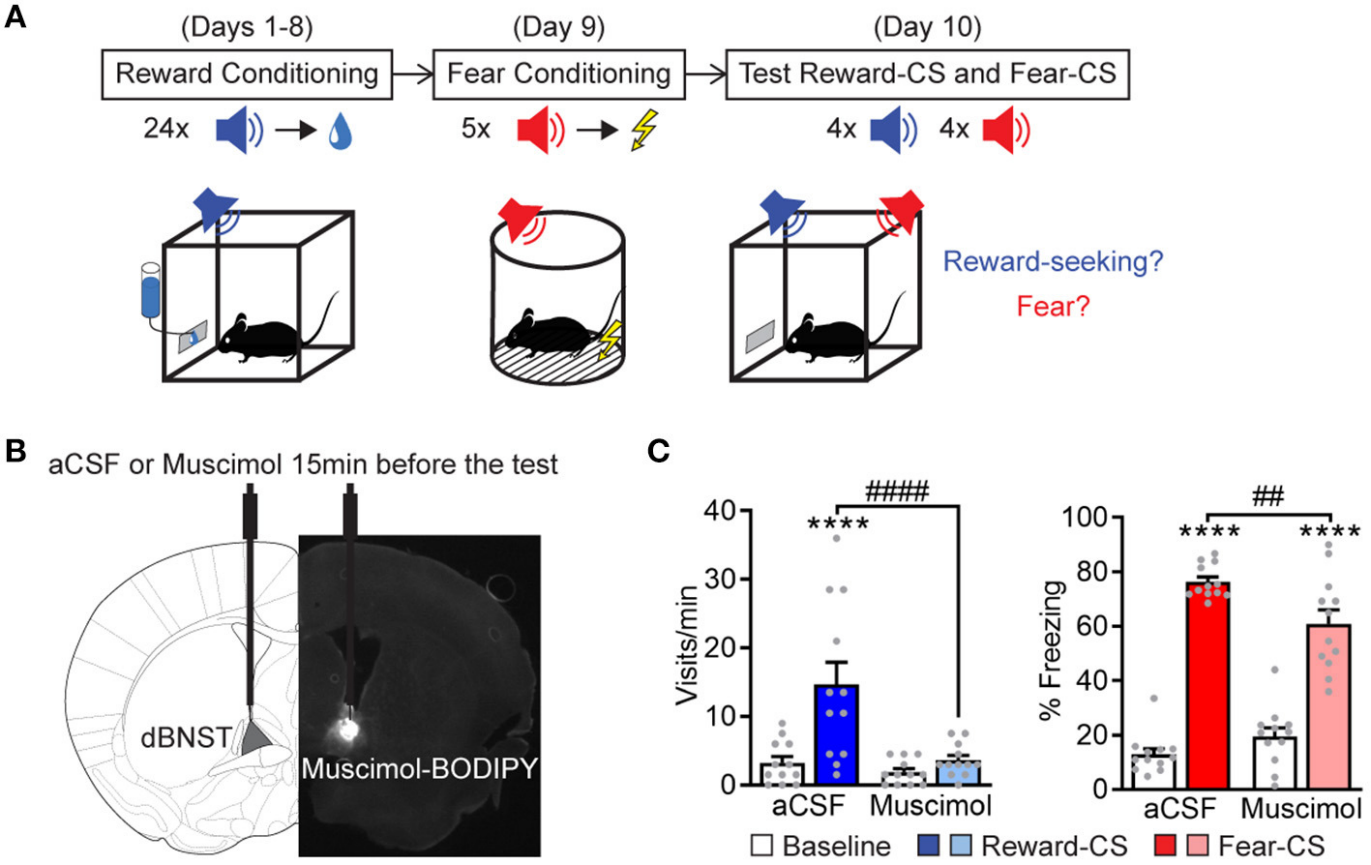
dBNST neurons are necessary for expressing fear and reward-related behaviors. **(A)** Experimental procedure. During reward conditioning (8 sessions), a reward-CS was paired with the delivery of a sucrose drop at the port of the cage. After successful learning, same animals underwent fear conditioning, which paired a different sound (fear-CS) with a footshock. During the test session, mice were re-exposed to the reward-CS and the fear-CS. We measured reward-seeking and fear behaviors. **(B)** A representative scheme of intra-dBNST infusion. About 15 min before the test, the mice had received an intra-dBNST infusion of aCSF or muscimol. After task completion, all the mice received an intra-dBNST infusion of muscimol-BODIPY for histological control, as depicted in the epifluorescent picture. **(C)** Left: dBNST inactivation before testing (Muscimol group, *n* = 12; compared to a CSF group, *n* = 12) abolished the expression of reward-seeking behavior (visits) during reward-CS re-exposure [two-way RM ANOVA: period (baseline vs. CS), *F*_(1, 22)_ = 16.34, *P* = 0.0005; group, *F*_(1, 22)_ = 10.75, *P* = 0.0034; interaction, *F*_(1, 22)_ = 8.786, *P* = 0.0072]. Right: Muscimol partially decreased freezing levels during fear-CS re-exposure [two-way RM ANOVA: period, *F*_(1, 22)_ = 366.9, *p* < 0.0001; group, *F*_(1, 22)_ = 1.365, *P* = 0.2553; interaction, *F*_(1, 22)_ = 15.98, *P* = 0.0006]. Data presented as mean + SEM. A *post-hoc* two-stage step-up method of Benjamini, Krieger, and Yekutieli: CS vs. baseline ****p < 0.0001; Muscimol vs. a CSF group ^##^*p* < 0.01 and ^####^*p* < 0.0001.

We adapted a combined Pavlovian reward and fear-conditioning paradigm (Shabel and Janak, 2009; Kargl et al., 2020) to exploit the advantages to differentiate fear and reward-related processes within the same defined circuit. The mice first learned a Pavlovian reward phase of the conditioning task (Figure 1A), in which an auditory-conditioned stimulus (Reward-CS) was paired with a reward, sucrose delivery inside a port. After successful learning, as assayed by increased port visits during Reward-CS (Supplementary Figure 1A), the mice learned the Pavlovian fear-conditioning phase, during which a different auditory cue (Fear-CS) was paired with an electric footshock (Supplementary Figure 1B). During the test phase, the mice were re-exposed to both the Reward-CS and Fear-CS, and we assessed the expression of correct affective response, i.e., freezing during Fear-CS and port visits during Reward-CS. Using this protocol, we probed if inactivating dBNST neurons would prevent the expression of those Pavlovian responses. So, we had infused muscimol bilaterally into dBNST 15 min before the testing (Figure 1B and Supplementary Figure 2A). This inactivation partially decreased fear responses during the Fear-CS and completely abolished reward-seeking behavior during the Reward-CS (Figure 1C), without provoking any change in mobility duration during the baseline (Supplementary Figure 2B), therefore preserving general exploratory behavior. We conclude that the BNST contributes to Pavlovian fear (Duvarci et al., 2009; Haufler et al., 2013; De Bundel et al., 2016; Bjorni et al., 2020) and reward-seeking behaviors (Shaham et al., 2003). However, the relatively small effect observed in freezing most likely reflects either redundancy in the circuitry involved in Pavlovian freezing (i.e., the amygdala) and/or ceiling effects in the experimental paradigm by overtraining with high-shock intensities.

### dBNST Neurons Encode Fear and Reward

**Figure 2:**
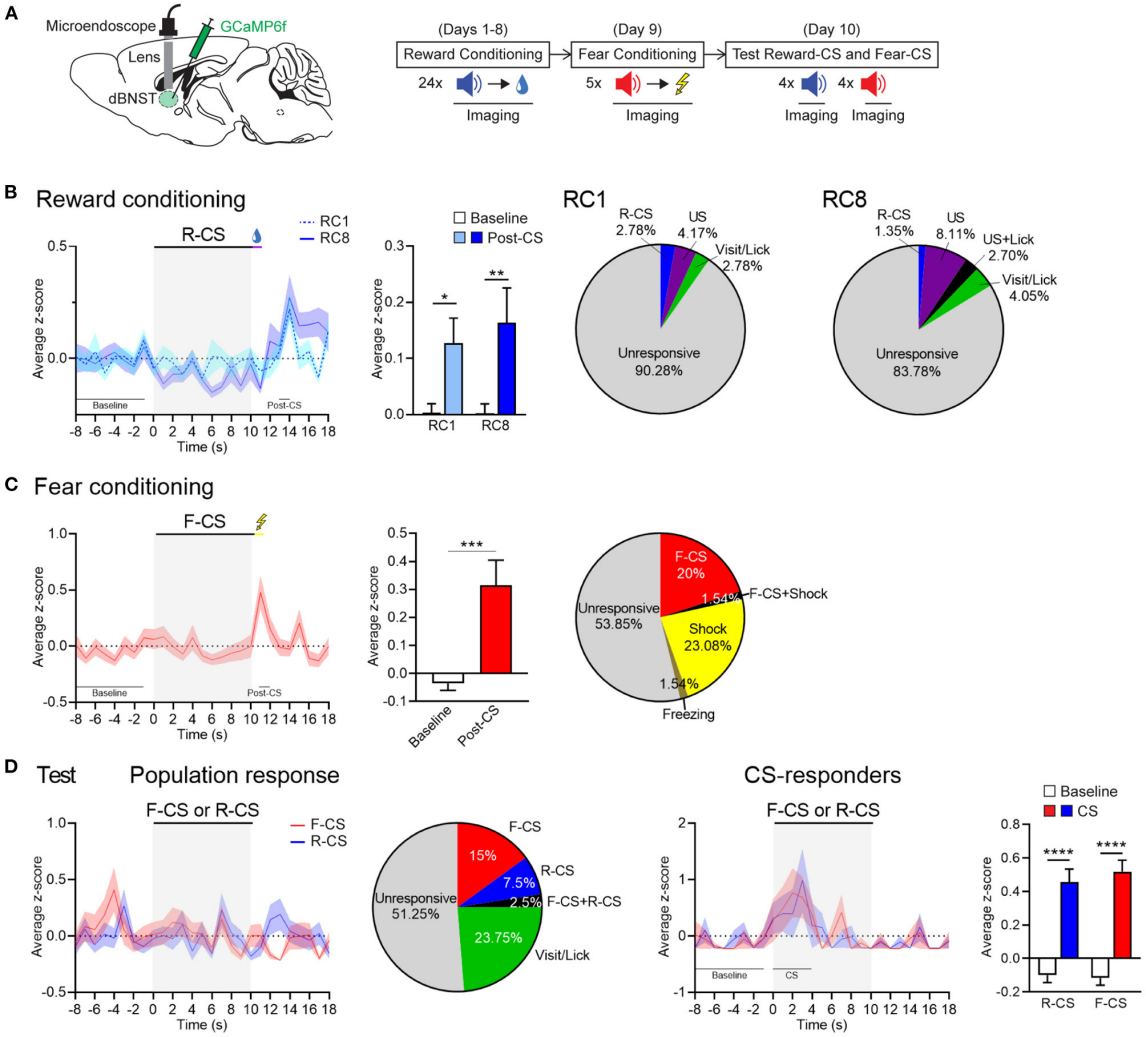
dBNST neurons encode fear and reward. **(A)** Left, schematic of dBNST injection and targeting for GCaMP imaging. Right, schematic of behavioral procedure combined with GCaMP imaging. **(B)** Left, PETH of population responses during the first (RC1) and last (RC8) sessions of reward conditioning. Middle, dBNST neurons increased their activity at maximum of the post-CS period (13–14 s) after reward delivery compared to the baseline (−8 to −1 s) at both RC1 and RC8 [two-way RM ANOVA: period, *F*_(1, 142)_ = 11.26, *P* = 0.0010; session, *F*_(1, 142)_ = 0.2222, *P* = 0.6381; interaction, *F*_(1, 142)_ = 0.1945, *P* = 0.6599]. Right, percentage of neurons responding to task stimuli and behaviors during RC1 (*n* = 72) and RC8 (*n* = 74; from 5 animals). **(C)** Left, PETH of population responses during fear conditioning. Middle, dBNST neurons increased their activity at maximum during the post-CS period (11–12 s) after shock compared to the baseline [two-tailed paired *t*-test: *t*_(62)_ = 3.524, *P* = 0.0008]. Right, percentage of neurons responding to task stimuli and behaviors (*n* = 65). **(D)** Left, PETH of population responses during the test and corresponding percentage of neurons responding to task stimuli and behaviors (*n* = 80). Right, PETH of CS-responders (R-CS: *n* = 13; F-CS: *n* = 15) and corresponding histogram summarizing average z-score during at maximum of the CS period (0–4 s) compared to the baseline (−8 to −1 s) [two-way RM ANOVA: period, *F*_(1, 26)_ = 72.65, *p* < 0.0001; CS, *F*_(1, 26)_ = 0.2094, *P* = 0.6510; interaction, *F*_(1, 26)_ = 0.3276, *P* = 0.5720]. Data presented as mean + SEM. Fisher's LSD *post-hoc* test, *p < 0.05, **p < 0.01, ***p < 0.001, and ****p < 0.0001. R-CS, reward-CS; F-CS, fear-CS.

We next performed deep brain calcium imaging in the freely moving mice to explore dBNST neuronal dynamics during Pavlovian fear and reward conditioning (Figure 2A). The mice were injected with GCaMP6f in the dBNST and implanted with a microendoscope above this structure (Supplementary Figure 3A). We recorded dBNST neuronal activity in the mice at different stages of the combined Pavlovian fear and reward conditioning during CSs and USs presentation (conditioning) or CSs alone (test) (Figure 2A). We identified between 65 and 80 neurons per session from 5 animals and extracted Ca^2+^ (calcium) events from their Ca^2+^ traces as a rise in Ca^2+^ signal > 5 s.d. (Supplementary Figure 3A, right, Supplementary Figure 4). We computed an event z-score and aligned it with the CS onset. Comparing the first and last sessions of reward conditioning, dBNST neuronal population showed a strong increase in Ca^2+^ events after reward delivery (Figure 2B, left). Further analysis detected cells responding to either the CS, US, and/or behavior onset based on a trial-averaged z-score difference of 1.65. This activity pattern indicated that individual neurons encoded the Reward-CS, the reward US, and/or the appetitive-conditioned behavior (visit and/or lick) (Figure 2B, right). During the fear-conditioning phase, the same population of dBNST neurons strongly increased its responding to the shock US (Figure 2C, left), with individual neurons responding to either the Fear-CS and/or the shock US (Figure 2C, right). During the test, a heterogeneous response profile emerged. Cells responded with the behavioral responses or selectively to either the Fear-CS or the Reward-CS (Figure 2D, left). CS-responder neurons showed an increase in activity during the first 4 s of CS presentation (Figure 2D, right). Thus, we uncovered differential response patterns in the dBNST that features valence-specific cells for either fear (CS) or reward (visit behavior). Of note, fear is encoded at the level of the CS, reward at the level of visits, as both events immediately precede and potentially bias the behavioral response (freezing, lick).

### dBNST-PVH and dBNST-PAG Circuits Differentially Encode Fear and Reward

**Figure 3:**
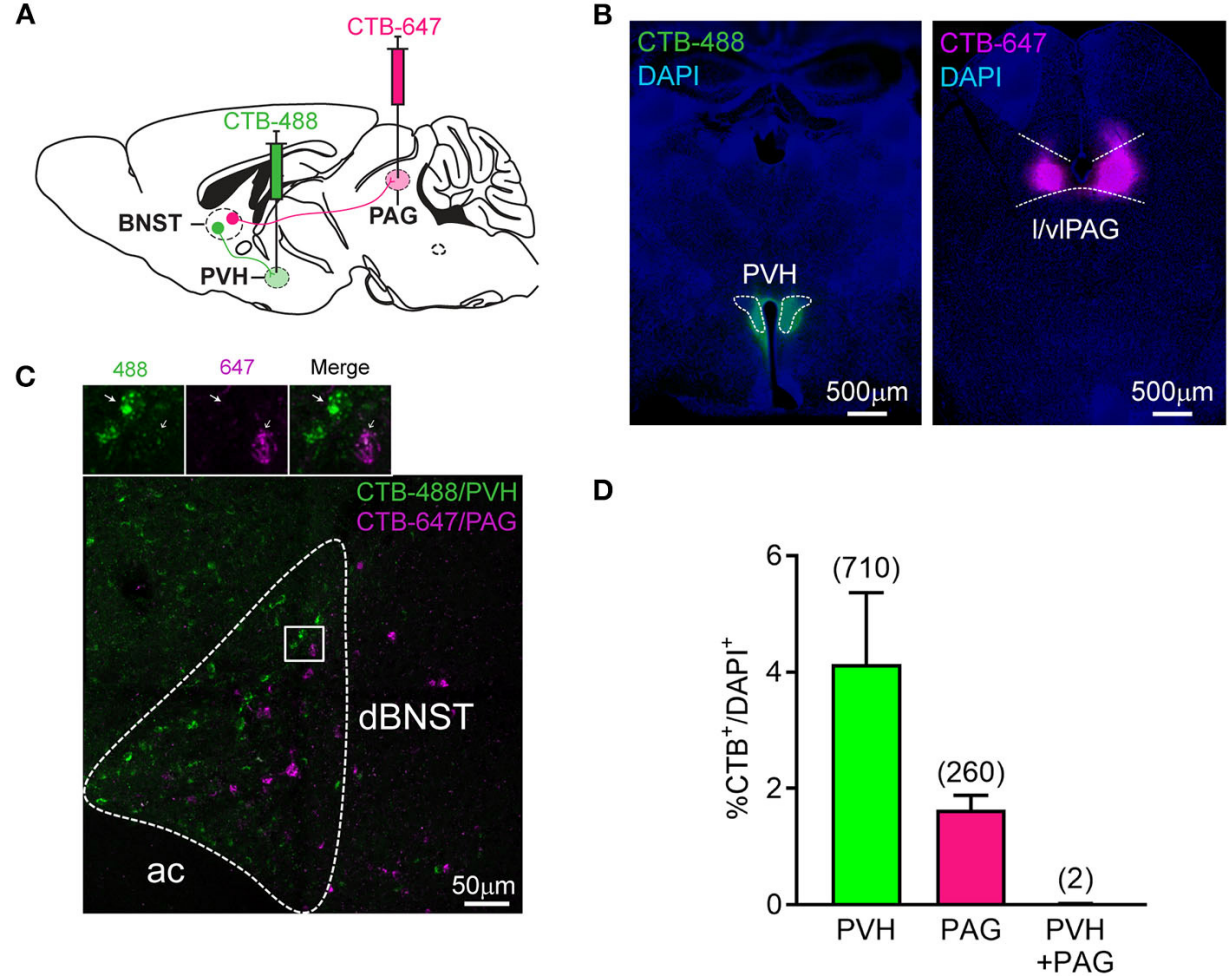
Segregated dBNST neurons project to PVH or PAG. **(A)** Schematic of the procedure. CTB-488 and CTB-647 were injected in the same mice in the PVH and l/vlPAG, respectively. A number of CTB retrogradely labeled neurons were measured in the dBNST. **(B)** Representative pictures of CTB-488 and CTB-647 injected in the PVH and l/vlPAG, respectively. **(C)** A representative picture of CTB-labeled neurons from PVH and l/vlPAG found in the dBNST. Most did not colocalize (see inset). Ac, anterior commissure. **(D)** CTB injected in PVH or l/vlPAG independently labeled a similar percentage of cells in the dBNST [*n* = 4 animals; two-tailed paired *t*-test: *t*_(3)_ = 1.891, *P* = 0.1550]. Number of co-labeled neurons (PVH + PAG, 0.01%) was lower than expected (0.08%) using a chi-square test (χ^2^ = 8.980, *P* = 0.0296) performed on the total number of cells counted from 4 animals (numbers in the parentheses in the graph; total number of CTB^–^/DAPI^+^ = 14,603). Data presented as mean + SEM.

Overall, our data demonstrate that dBNST encodes both fear and reward states with specific neuronal populations differentially tuned to valence and feature (CS, behavior) of affective processing. We next examined whether these different response types map to different dBNST output channels. There are several candidate downstream regions, which are known to regulate affective responding, in particular, fear responses via PAG or stress, energy homeostasis, and feeding via PVH (LeDoux, 2000; Williams et al., 2001; Herman et al., 2003; Xu et al., 2019). Thus, we hypothesized that dBNST neurons projecting to PVH and those projecting to PAG subserved distinct functional responses. So, we injected the same animals with cholera toxin subunit B (CTB) conjugated with different fluorophores into the PVH and the lateral/ventrolateral PAG [l/vlPAG, key subregion gating freezing behavior (Tovote et al., 2016)] and counted back-labeled projecting neurons in the dBNST (Figures 3A,B). Approximately, 4% of dBNST cells projected to PVH and 2% of dBNST cells to l/vlPAG. Most projections did not co-localize (Figures 3C,D), raising the possibility that these outputs represent different functional channels. These results also indicate that we can label and explore each circuit independently using this segregation.

**Figure 4:**
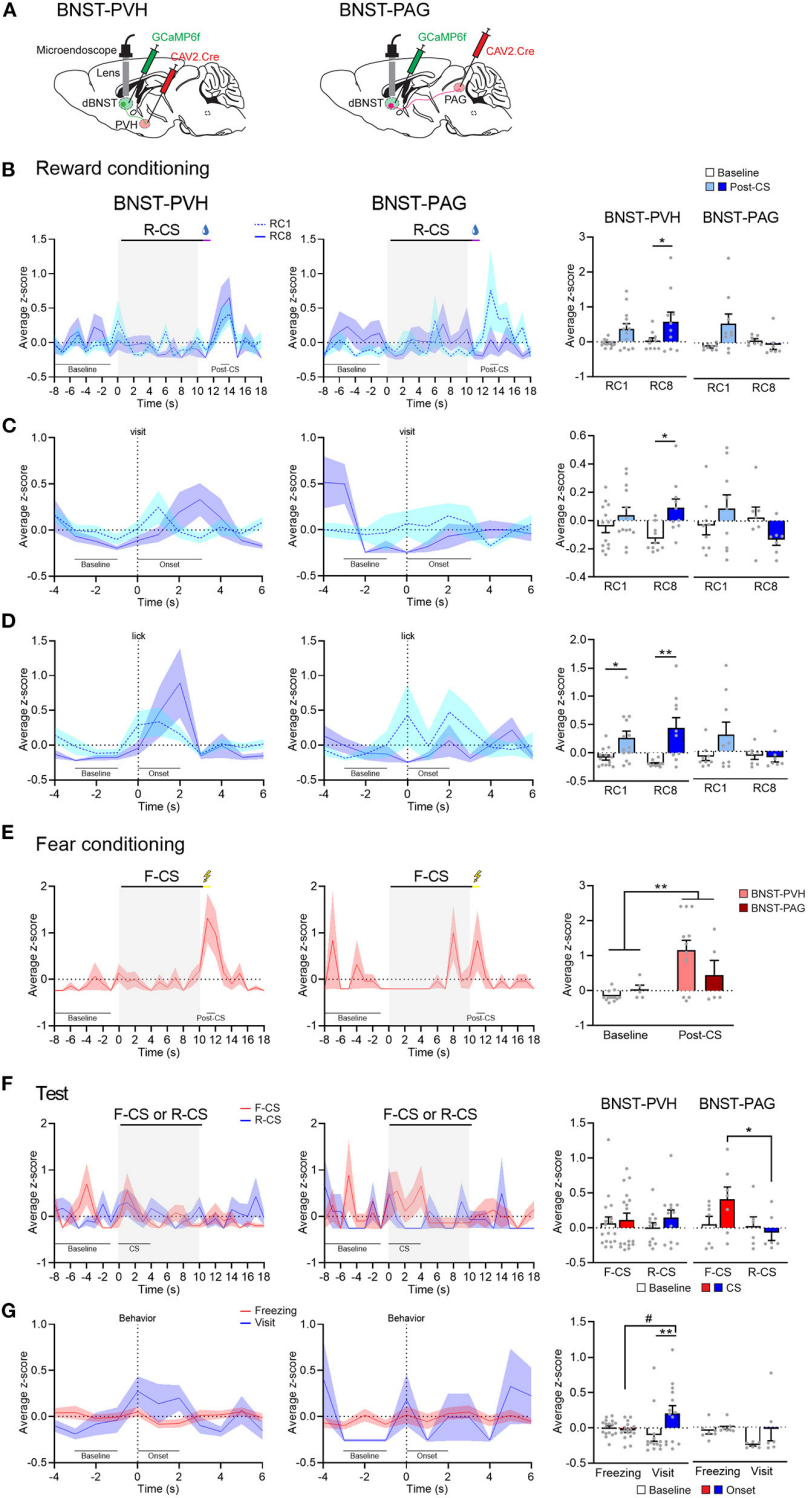
BNST-PVH and BNST-PAG pathways differentially encode fear and reward. **(A)** Schematic of injections and targeting for GCaMP imaging in BNST-PVH (left) or -PAG (right) projecting neurons. **(B)** Left, PETH of population responses during the first (RC1) and last (RC8) sessions of reward conditioning in BNST-PVH (RC1: *n* = 13; RC8: *n* = 10; from 3 animals) and BNST-PAG (RC1: *n* = 9; RC8: *n* = 7; from 3 animals) groups. Right, BNST-PVH neurons increased their activity at maximum of the post-CS period (13–14 s) after reward delivery compared to the baseline (−8 to −1 s) at both RC1 and RC8 [two-way RM ANOVA: period, *F*_(1, 21)_ = 7.736, *P* = 0.0112; session, *F*_(1, 21)_ = 1.159, *P* = 0.2940; interaction, *F*_(1, 21)_ = 0.1607, *P* = 0.6925], but not BNST-PAG neurons [two-way RM ANOVA: period, *F*_(1, 14)_ = 2.067, *P* = 0.1725; session, *F*_(1, 14)_ = 1.784, *P* = 0.2030; interaction, *F*_(1, 14)_ = 4.408, *P* = 0.0544]. **(C)** Left, PETH of population responses aligned with the visit onset. Right, BNST-PVH neurons increased their activity to maximum after the visit onset (0–3 s) compared to the baseline (−3 to −1 s) [two-way RM ANOVA: period, *F*_(1, 21)_ = 5.771, *P* = 0.0256; session, *F*_(1, 21)_ = 0.3022; *P* = 0.5883; interaction, *F*_(1, 21)_ = 1.245, *P* = 0.2772] but not BNST-PAG neurons [two-way RM ANOVA: period, *F*_(1, 14)_ = 0.04002, *P* = 0.8443, session, *F*_(1, 14)_ = 1.819, *P* = 0.1989; interaction, *F*_(1, 14)_ = 2.263, *P* = 0.1548]. **(D)** Left, PETH of population responses aligned with the lick onset. Right, BNST-PVH neurons increased their activity at the maximum period after the lick onset (0–2 s) compared to the baseline (−3 to −1 s) [two-way RM ANOVA: period, *F*_(1, 21)_ = 16.75, *P* = 0.0005; session, *F*_(1, 21)_ = 0.1231, *P* = 0.7292; interaction, *F*_(1, 21)_ = 1.440, *P* = 0.2436] but not BNST-PAG neurons [two-way RM ANOVA: period, *F*_(1, 14)_ = 1.503, *P* = 0.2404; session, *F*_(1, 14)_ = 2.209, *P* = 0.1593; interaction, *F*_(1, 14)_ = 2.006, *P* = 0.1786]. **(E)** Left, PETH of population responses during fear conditioning in BNST-PVH (*n* = 12) and BNST-PAG (*n* = 5) groups. Right, both BNST-PVH and BNST-PAG groups increased their activity at maximum of the post-CS period (11–12 s) after shock compared to the baseline [two-way RM ANOVA: period, *F*_(1, 15)_ = 9.159, *P* = 0.0085; group, *F*_(1, 15)_ = 1.088, *P* = 0.3135; interaction, *F*_(1, 15)_ = 2.475, *P* = 0.1365]. **(F)** Left, PETH of population responses during test. Right, BNST-PAG neurons increased their activity during F-CS trials (*n* = 7) compared to R-CS (*n* = 6) [two-way RM ANOVA: CS, *F*_(1, 11)_ = 6.885, *P* = 0.0237; period, *F*_(1, 11)_ = 0.5879, *P* = 0.4594; interaction, *F*_(1, 11)_ = 1.727, *P* = 0.2156] but not BNST-PVH neurons (F-CS: *n* = 17; R-CS: *n* = 12) [two-way RM ANOVA: CS, *F*_(1, 27)_ = 0.02511, *P* = 0.8753; period, *F*_(1, 27)_ = 0.6768, *P* = 0.4179; interaction, *F*_(1, 27)_ = 0.1849, *P* = 0.6706]. **(G)** Left, PETH of population responses aligned with the behavior onset. Right, BNST-PVH neurons increased their activity to maximum after the visit onset (0–2 s; *n* = 14) compared to the baseline (−3 to −1 s) and the freezing onset (*n* = 17) [two-way RM ANOVA: period, *F*_(1, 29)_ = 4.150, *P* = 0.0509; behavior, *F*_(1, 29)_ = 0.9202, *P* = 0.3453; interaction, *F*_(1, 29)_ = 7.172, *P* = 0.0121] but not BNST-PAG neurons (visit: *n* = 6; freezing: *n* = 7) [two-way RM ANOVA: period, *F*_(1, 11)_ = 3.304, *P* = 0.0964; behavior, *F*_(1, 11)_ = 1.604, *P* = 0.2315; interaction, *F*_(1, 11)_ = 1.268, *P* = 0.2841]. Data presented as mean + SEM. Fisher's LSD *post-hoc* test, */^#^*p* < 0.05 and **p < 0.01.

Given the segregation of dBNST projections to PVH and PAG, we next queried the functional relevance of this anatomical difference. We speculated that these pathways encoded different phases of fear and reward Pavlovian learning. The mice were injected with a canine virus (CAV) expressing Cre in the PVH or l/vlPAG and with an AAV expressing Cre-dependent GCaMP6f in the dBNST (Figure 4A). Using this approach, we could record specific dBNST projectors to PVH or l/vlPAG, respectively (Supplementary Figures 3B,C). Comparing the first and last sessions of reward conditioning, we observed a strong increase in Ca^2+^ events a few seconds after reward delivery in dBNST-PVH neurons (Figure 4B), but not dBNST-PAG neurons, similar to the overall dBNST population (Figure 2B). Interestingly, z-scores and raw traces in neuronal activity (Supplementary Figure 5) showed a peak in dBNST-PVH neurons Ca^2+^ events aligned within a few seconds of a port visit (Figure 4C) and the lick onset (Figure 4D). During fear conditioning, both dBNST-PVH and dBNST-PAG populations reacted strongly to the shock US (Figure 4E), suggesting that both pathways encode natural negative stimuli. This pattern was even more pronounced during the test phase. Here, dBNST-PAG neurons responded more strongly to fear (CS trials) compared to reward (CS trial; Figure 4F). However, the maximal dBNST-PVH neuron Ca^2+^ events were aligned with reward (visit onset), but not fear (freezing; Figure 4G). Collectively, these response patterns revealed a dissociation between the activity of those circuits in the encoding aspects of fear and rewarding (see also Supplementary Figure 6). Based on these results, we conclude that dBNST-PVH neurons are tuned to process reward states, whereas dBNST-PAG neurons are tuned to process fear. In addition, the dBNST-PAG circuit encodes the Pavlovian CS, while the dBNST-PVH encodes the conditioned reward-seeking behavior.

### dBNST-PVH and dBNST-PAG Circuits Differentially Gate Fear and Reward Responses

**Figure 5:**
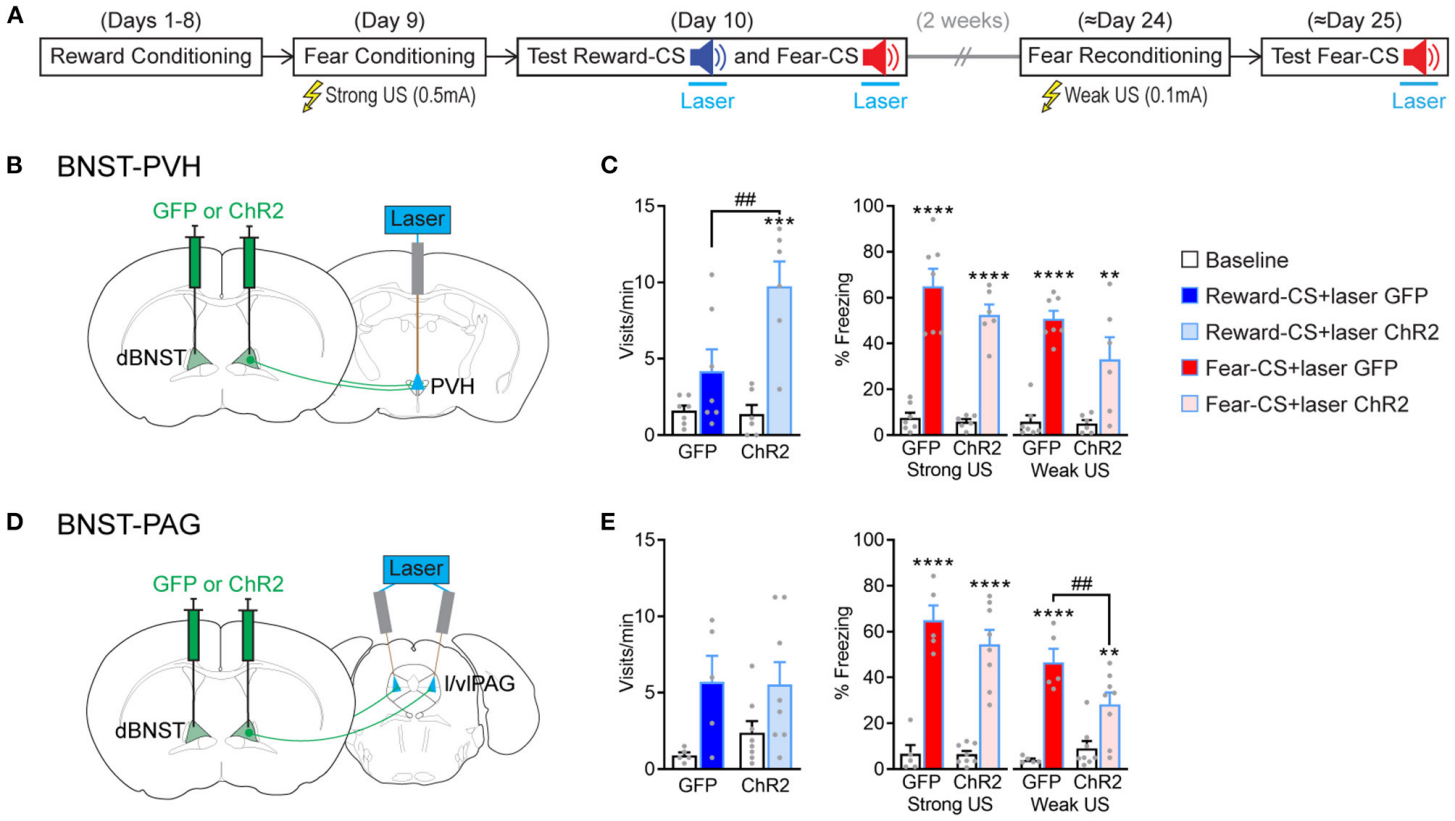
BNST-PVH and BNST-PAG circuits differently gate fear and reward responses. **(A)** Experimental procedure. The mice underwent the combined reward and fear-conditioning protocol. During the test, the laser was activated during the 10-s duration of Reward-CS and Fear-CS presentations. About 2 weeks later, the mice were reconditioned to the Fear-CS using a weak US (0.1 mA) and tested the next day with Fear-CS presentations, coupled with laser activation. **(B)** Schematic of the BNST-PVH targeting strategy for optogenetic manipulations. Briefly, an AAV construct carrying a GFP or ChR2 was bilaterally injected in dBNST. One optic fiber was placed above the PVH for further optogenetic activation of dBNST-PVH projections. **(C)** Behavioral results of test sessions. Left, during Reward-CS, the mice of the BNST-PVH ChR2 group (*n* = 6) increased their visits compared to the GFP control group (*n* = 7) [two-way RM ANOVA: group, *F*_(1, 11)_ = 5.512, *P* = 0.0386; period, *F*_(1, 11)_ = 23.89, *P* = 0.0005; interaction, *F*_(1, 11)_ = 6.730, *P* = 0.0249]. Right, during Fear-CS, all the mice froze more during the sound compared to the baseline in every condition [Strong US, two-way RM ANOVA: period, *F*_(1, 11)_ = 131.5, *p* < 0.0001; group, *F*_(1, 11)_ = 1.882, *P* = 0.1975; interaction, *F*_(1, 11)_ = 1.409, *P* = 0.2603; Weak US, two-way RM ANOVA: period, *F*_(1, 11)_ = 66.07, *p* < 0.0001; group, *F*_(1, 11)_ = 2.577, *P* = 0.1367; interaction, *F*_(1, 11)_ = 3.505, *P* = 0.0880]. (**D**) Schematic of BNST-PAG targeting strategy, as described above, except that two optic fibers were placed above the l/vlPAG with a 10° angle. **(E)** Left, during Reward-CS, the mice in the BNST-PAG ChR2 group (*n* = 8) and the GFP control group (*n* = 5) increased their visits during the sound [two-way RM ANOVA: period, *F*_(1, 11)_ = 8.804, *P* = 0.0128; group, *F*_(1, 11)_ = 0.3228, *P* = 0.5813; interaction, *F*_(1, 11)_ = 0.3855, *P* = 0.5473]. Right, during Fear-CS, analyses restricted to each US situation revealed that the BNST-PAG ChR2 group froze less than the control GFP group in the weak US condition [Strong US, two-way RM ANOVA: period, *F*_(1, 11)_ = 143.3, *p* < 0.0001; group, *F*_(1, 11)_ = 0.9169, *P* = 0.3589; interaction, *F*_(1, 11)_ = 1.335, *P* = 0.2724; Weak US, two-way RM ANOVA: period, *F*_(1, 11)_ = 65.96, *p* < 0.0001, group, *F*_(1, 11)_ = 1.689, *P* = 0.2203; interaction, *F*_(1, 11)_ = 9.621, *P* = 0.0101]. Data presented as mean + SEM. A *post-hoc* two-stage step-up method of Benjamini, Krieger, and Yekutieli: CS vs. baseline **p < 0.01, ***p < 0.001, and ****p < 0.0001; ChR2 vs. GFP group ^##^*p* < 0.01.

As dBNST-PVH and dBNST-PAG activity is biased for reward or fear, respectively, we wondered how these circuits then control affective responding. So, we probed whether optogenetic activation of those circuits modulated reward and fear behavioral responses. We implanted optic fibers above PVH or l/vlPAG and bilaterally injected the mice with an AAV construct either carrying GFP or ChR2 into the dBNST for subsequent optogenetic activation (Figures 5B,D and Supplementary Figure 7). We utilized our Pavlovian reward and fear conditioning and applied optogenetic stimulation throughout Reward-CS and Fear-CS re-exposure during the test phase (Figure 5A). Optogenetic activation of dBNST-PVH increased the port visit rate during the Reward-CS in the ChR2 group (Figure 5C, left). The same manipulation in the dBNST-l/vlPAG circuit did not change the rate of this reward-seeking behavior (Figure 5E, left). In both experiments, optogenetic manipulation did not change the time spent visiting the port (Supplementary Figure 8). For fear conditioning, we used two different settings. The typical setting with 0.5 mAUS intensity, as in our previous experiments (Figures 1, 2, 4), did not significantly change during stimulation of either the dBNST-PVH circuit (Figure 5C, right) or the dBNST-l/vlPAG circuit (Figure 5E, right). We suspect a ceiling effect may explain these results.

We, therefore, reconditioned the mice 2 weeks later to the Fear-CS using a low-intensity setting (weak US, 0.1 mA) and tested them again the next day. This modified procedure unmasked a partial decrease in freezing behavior during optogenetic activation of the dBNST-l/vlPAG circuit (Figure 5E, right), which did not reach significance by activating the dBNST-PVH circuit (Figure 5C, right). These data reveal that activating dBNST-PVH and dBNST-l/vlPAG circuits can preferentially modulate either reward or fear states, respectively, which aligns with their inherent activity tuning preference (Figure 4). However, this seemingly occurs by increasing reward and decreasing fear responding, which produces a positively valenced net effect. Overall, dBNST is required to induce fear and reward responses (Figure 1). Thus, dBNST-PVH/PAG circuits subserve distinct functional and modulatory roles. These roles are independent of overall dBNST inactivation effects and function, underlining the complex functional organization of limbic nuclei.

## Discussion

The same brain region can encode opposite affective states (Calder et al., 2001; Lammel et al., 2014; Namburi et al., 2016). Here, we show that dBNST neurons that project to specific brain outputs differentially encode specific affective features of Pavlovian fear and reward conditioning. Ultimately, these distinct circuits bias a behavioral outcome toward overall positive-valenced behavioral expressions (Supplementary Figure 9).

Inactivating dBNST neurons partially reduced Pavlovian fear responses and fully abolished reward-seeking behavior. Our data advance the proposal that the BNST plays a general role in Pavlovian fear (Duvarci et al., 2009; Haufler et al., 2013; De Bundel et al., 2016; Bjorni et al., 2020) and in reward (Jennings et al., 2013a; Kim et al., 2013; Giardino et al., 2018; Girven et al., 2020). We identified here that dBNST circuitry is a key node to process both Pavlovian fear and reward responses. We exploited a combined Pavlovian task to assess both fear and reward processing in the same animal (Shabel and Janak, 2009; Kargl et al., 2020). In this task, mice learned sequentially a reward conditioning task, a fear conditioning task, following testing to both Reward-CS and Fear-CS in the reward context. Although this type of settings could bias responding toward appetitive valence *per se*, we note that strong fear responding appeared in control groups expressed in high levels (Figures 1, 5). Thus, our findings further support the role of BNST in phasic Pavlovian fear (Duvarci et al., 2009; Haufler et al., 2013; De Bundel et al., 2016; Bjorni et al., 2020). Whereas, previous studies showed a specific role for the BNST in sustained fear but not in phasic fear to a single CS (Davis et al., 2010), recent studies have uncovered a role for the BNST in discriminative phasic fear when another CS (CS-, not associated with the shock) is present during training (Duvarci et al., 2009; De Bundel et al., 2016). Our data reinforce the hypothesis of a role of BNST in discriminative fear, here in a condition of a discriminative fear-reward learning. The fact that dBNST inactivation only partially reduces fear responses is in line with the view that the BNST functions as a secondary site for Pavlovian fear states in conjunction with the amygdala (Poulos et al., 2010), the key brain structure for fear learning (LeDoux, 2000; Tovote et al., 2015). In parallel, our Pavlovian paradigm had a key advantage to assess the role of BNST in reward processing. Typically, BNST functions were examined using drug-related behaviors (Erb et al., 2001; Shaham et al., 2003), feeding (Jennings et al., 2013b; Giardino et al., 2018), or optogenetically induced conditioned place preference or intracranial self-stimulation (Jennings et al., 2013a; Kim et al., 2013; Giardino et al., 2018; Girven et al., 2020), but very few studies directly explored conditioned behaviors using natural rewards. Harnessing the advantages of our Pavlovian task, we could establish that the dBNST is critical to expressing conditioned reward-seeking behaviors. As food or operant conditioning (Dumont et al., 2005) can increase synaptic plasticity in the vBNST, our findings highlight the importance of the BNST in reward-seeking behaviors. Our findings position the BNST as a key processing node in reward circuitry with the VTA, the amygdala, the nucleus accumbens among others (Everitt et al., 1999).

Using Ca^2+^ imaging, we observed broad excitation of BNST neurons (independent of their projections) in response to natural positive and negative USs (shock and sucrose), which extends findings of increased BNST Ca^2+^ signals in response to positive and negative odor stimuli (Giardino et al., 2018). We identified at the local level two submodules that have independent modulatory functions. Specifically, we found that dBNST neurons, particularly those that project to the PVH, are strongly activated by reward behaviors (visits and/or licks) following reward US or during Reward-CS re-exposure. We propose that PVH-projecting dBNST neurons encode conditioned reward behaviors, as two types of conditioned responses required the CS (visit) or the US (lick) (Holland, 1977). This projection could also encode specific reward state features when it is present and consumed during reward-conditioning sessions. Consistent with our hypothesis that the dBNST-PVH pathway modulates reward-seeking behaviors, we observed that, activating this circuit by optogenetics, the mice sought more the reward by visiting more the port. Although the PVH is well-known to be the starting point of the hypothalamic-pituitary-adrenal (HPA) axis and is essential for orchestrating stress responses (Herman et al., 2003), this structure is also involved in food consumption (Leibowitz et al., 1981), and reinforced food-seeking (Atasoy et al., 2012). We demonstrate the functional relevance of the direct projection between BNST and PVH in Pavlovian reward learning. Since the mice increased their visits in absence of food with PVH-projecting BNST neuron activation, we propose this behavior can underlie compulsive reward-seeking behavior, which is the key feature of drug addiction and eating disorders (Everitt and Robbins, 2005; American Psychiatric Association, 2013). Similar compulsive reward-seeking behaviors are controlled by the neural circuit between the central amygdala and the BNST (Kim et al., 2018) or by hypothalamic Agrp neurons whose one of the main projection sites is the PVH (Dietrich et al., 2015). Calcium imaging experiments by others showed that corticotrophin-releasing hormone (CRH) PVH neurons are activated by negative stimuli, inhibited by positive stimuli, produce conditioned place aversion, and contribute to reward-induced stress modulation, probably through GABAergic innervation from BNST (Kim et al., 2019; Yuan et al., 2019). Thus, we propose that an inhibitory pathway between BNST and PVH biases the emotional experience toward a positive valence.

Our results further suggest that dBNST neurons projecting to PAG encode and modulate negative features of the emotional experience (CS and behavior) but lead to an overall behavioral change that reflects a decreased negative valence. Indeed, our results show that Fear-CS activates PAG-projecting dBNST neurons. However, optogenetic activation of this projection decreases freezing behaviors. This finding implies that BNST-projecting neurons to PAG encode Fear-CS, as modulating freezing is consistent with the role of PAG, especially the l/vlPAG, in freezing control (Tovote et al., 2016). Conversely, in our hands, l/vlPAG-projecting dBNST neurons appear to have a lesser role in encoding or controlling reward learning. However, a recent study has uncovered the role of GABAergic vlPAG neurons and their dBNST inputs in regulating feeding (Hao et al., 2019). Those discrepancies may be explained by a more complex function and cognitive processes in discriminatory Pavlovian conditioning [vs. basic feeding (Hao et al., 2019)]. Further investigations are needed to elucidate the whole picture of dBNST, PVH, and l/vlPAG circuitries in regulating fear and reward. We note that, although the tracing experiment revealed segregated and rather specific dBNST projections to PVH and PAG, it is possible that some collateral projections terminate in other brain areas (e.g., ventral BNST, amygdala, and many others), which could contribute, in part, to the observed optogenetic effects (albeit that coactivation of such projections can be assumed to be rather limited).

Taken together, neural activity in the dBNST encodes both fear and reward states. However, the Ca^2+^ activity profiles dissociated different features of the Pavlovian paradigm: dBNST neurons projecting to the PVH are activated with the expression of Pavlovian reward conditioned behaviors (visits and licks), whereas dBNST neurons projecting to the l/vlPAG are excited by the Pavlovian Fear-CS. We demonstrate a dichotomy between valence (fear vs. reward) and Pavlovian features (stimulus vs. behavior). Identifying neural correlates of opposing emotions often utilizes discriminating between positive vs. negative valence (Lammel et al., 2014; Namburi et al., 2016), or active vs. passive behaviors (Tovote et al., 2015). Here, our data advance another dichotomy in discriminating the neural encoding of opposing fear vs. reward emotional states: Pavlovian stimulus vs. behavior. While BNST circuitry represents both fear (BNST-PAG circuit) and reward signals (BNST-PVH circuit), they promote an overall positive response bias in both domains (Supplementary Figure 9). A similar dichotomy in roles can exist in anteroventral BNST circuits with either PVH or PAG to modulate HPA axis activation or behavioral immobility responses in behavioral tests measuring stress (Johnson et al., 2016). Thus, our findings suggest that BNST neurons have much broader roles in regulating various aspects of negative and positive emotional experiences.

Collectively, we found that BNST circuitry contributes to encoding opposite Pavlovian elements of learning (fear CS vs. reward behaviors), which bias the emotional experience and modulate behavioral outcomes toward positive valence, either by attenuating negative fear states or by intensifying positive reward-related states (Supplementary Figure 9). This bias to the same positive valence supported by distinct neuronal populations advances our knowledge of studying opposing emotions, in contrast to switching between positive and negative valences (Paton et al., 2006; Shabel and Janak, 2009; Cohen et al., 2012; Lammel et al., 2012; Beyeler et al., 2016; Kim et al., 2016). This positive response bias in fear and reward supports the hypothesis of reward-induced coping strategies to manage stress (Kim et al., 2019; Yuan et al., 2019). Our data highlight BNST circuitry as key networks that may underlie positive valence bias in normal and pathological conditions. Thus, we propose that the dBNST-PAG/PVH network controls reward-seeking under risk. This response strategy could support the emergence of compulsive reward-seeking behaviors while attenuating the effects of negative stimuli, affects, or consequences. Overall, we speculate that overactive dBNST-PAG/PVH circuitry may drive maladaptive stress-induced behaviors like in drug-seeking or eating disorders (Everitt and Robbins, 2005; American Psychiatric Association, 2013).

## Data Availability Statement

The raw data supporting the conclusions of this article will be made available by the authors, without undue reservation.

## Ethics Statement

All animal care and behavioral tests were conducted in agreement with the Austrian (BGBl nr. 501/1988, idF BGBl I No. 162/2005) and European (Directive 86/609/EEC of 24 November 1986, European Community) legislation on animal experimentation and covered by the license M58/002220/2011/9.

## Author Contributions

NK and WH conceived the project. NK, SA, and MH performed experiments and data analysis. NK, SA, and WH wrote the manuscript. All authors contributed to the article and approved the submitted version.

## Funding

WH was supported by a grant from the European Community's Seventh Framework Programme (FP/2007–2013)/ERC grant agreement No. 311701, the Research Institute of Molecular Pathology (IMP), Boehringer Ingelheim, and the Austrian Research Promotion Agency (FFG). NK was supported by long-term postdoctoral fellowships from Fyssen Foundation, EMBO (Grant No. 1214–2012), and Marie Curie Actions (Grant No. 331015).

## Conflict of Interest

The authors declare that the research was conducted in the absence of any commercial or financial relationships that could be construed as a potential conflict of interest.

## Publisher's Note

All claims expressed in this article are solely those of the authors and do not necessarily represent those of their affiliated organizations, or those of the publisher, the editors and the reviewers. Any product that may be evaluated in this article, or claim that may be made by its manufacturer, is not guaranteed or endorsed by the publisher.

## References

[B1] American Psychiatric Association (2013). Diagnostic and Statistical Manual of Mental Disorders, 5th Edn. Washington, DC: American Psychiatric Association. 10.1176/appi.books.9780890425596

[B2] Atasoy D. Betley J. N. Su H. H. Sternson S. M. (2012). Deconstruction of a neural circuit for hunger. Nature 488, 172–177. 10.1038/nature11270 22801496 PMC3416931

[B3] Avery S. N. Clauss J. A. Blackford J. U. (2016). The human BNST: functional role in anxiety and addiction. Neuropsychopharmacology 41, 126–141. 10.1038/npp.2015.185 26105138 PMC4677124

[B4] Beyeler A. Namburi P. Glober G. F. Simonnet C. Calhoon G. G. Conyers G. F.. (2016). Divergent routing of positive and negative information from the amygdala during memory retrieval. Neuron 90, 348–361. 10.1016/j.neuron.2016.03.004 27041499 PMC4854303

[B5] Bjorni M. Rovero N. G. Yang E. R. Holmes A. Halladay L. R. (2020). Phasic signaling in the bed nucleus of the stria terminalis during fear learning predicts within- and across-session cued fear expression. Learn. Mem. 27, 83–90. 10.1101/lm.050807.119 32071254 PMC7029722

[B6] Calder A. J. Lawrence A. D. Young A. W. (2001). Neuropsychology of fear and loathing. Nat. Rev. Neurosci. 2, 352–363. 10.1038/35072584 11331919

[B7] Ch'ng S. Fu J. Brown R. M. McDougall S. J. Lawrence A. J. (2018). The intersection of stress and reward: BNST modulation of aversive and appetitive states. Prog. Neuropsychopharmacol. Biol. Psychiatry 87, 108–125. 10.1016/j.pnpbp.2018.01.005 29330137

[B8] Cohen J. Y. Haesler S. Vong L. Lowell B. B. Uchida N. (2012). Neuron-type-specific signals for reward and punishment in the ventral tegmental area. Nature 482, 85–88. 10.1038/nature10754 22258508 PMC3271183

[B9] Cullinan W. E. Herman J. P. Watson S. J. (1993). Ventral subicular interaction with the hypothalamic paraventricular nucleus: evidence for a relay in the bed nucleus of the stria terminalis. J. Comp. Neurol. 332, 1–20. 10.1002/cne.903320102 7685778

[B10] Davis M. Walker D. L. Miles L. Grillon C. (2010). Phasic vs. sustained fear in rats and humans: role of the extended amygdala in fear vs. anxiety. Neuropsychopharmacology 35, 105–135. 10.1038/npp.2009.109 19693004 PMC2795099

[B11] De Bundel D. Zussy C. Espallergues J. Gerfen C. R. Girault J.-A. Valjent E. (2016). Dopamine D2 receptors gate generalization of conditioned threat responses through mTORC1 signaling in the extended amygdala. Mol. Psychiatry 21, 1545–1553. 10.1038/mp.2015.210 26782052 PMC5101541

[B12] Dietrich M. O. Zimmer M. R. Bober J. Horvath T. L. (2015). Hypothalamic Agrp neurons drive stereotypic behaviors beyond feeding. Cell 160, 1222–1232. 10.1016/j.cell.2015.02.024 25748653 PMC4484787

[B13] Dong H.-W. Swanson L. W. (2004). Organization of axonal projections from the anterolateral area of the bed nuclei of the stria terminalis. J. Comp. Neurol. 468, 277–298. 10.1002/cne.10949 14648685

[B14] Dong H.-W. Swanson L. W. (2006). Projections from bed nuclei of the stria terminalis, anteromedial area: cerebral hemisphere integration of neuroendocrine, autonomic, and behavioral aspects of energy balance. J. Comp. Neurol. 494, 142–178. 10.1002/cne.20788 16304685 PMC2563961

[B15] Dong H. W. Petrovich G. D. Swanson L. W. (2001). Topography of projections from amygdala to bed nuclei of the stria terminalis. Brain Res. Brain Res. Rev. 38, 192–246. 10.1016/s0165-0173(01)00079-0 11750933

[B16] Dumont E. C. Mark G. P. Mader S. Williams J. T. (2005). Self-administration enhances excitatory synaptic transmission in the bed nucleus of the stria terminalis. Nat. Neurosci. 8, 413–414. 10.1038/nn1414 15735642 PMC4011824

[B17] Duvarci S. Bauer E. P. Paré D. (2009). The bed nucleus of the stria terminalis mediates inter-individual variations in anxiety and fear. J. Neurosci. 29, 10357–10361. 10.1523/JNEUROSCI.2119-09.2009 19692610 PMC2741739

[B18] Erb S. Shaham Y. Stewart J. (2001). Stress-induced relapse to drug seeking in the rat: role of the bed nucleus of the stria terminalis and amygdala. Stress 4, 289–303. 10.3109/10253890109014753 22432148

[B19] Everitt B. J. Parkinson J. A. Olmstead M. C. Arroyo M. Robledo P. Robbins T. W. (1999). Associative processes in addiction and reward. The role of amygdala-ventral striatal subsystems. Ann. N. Y. Acad. Sci. 877, 412–438. 10.1111/j.1749-6632.1999.tb09280.x 10415662

[B20] Everitt B. J. Robbins T. W. (2005). Neural systems of reinforcement for drug addiction: from actions to habits to compulsion. Nat. Neurosci. 8, 1481–1489. 10.1038/nn1579 16251991

[B21] Fanselow M. S. (1980). Conditioned and unconditional components of post-shock freezing. Pavlov. J. Biol. Sci. 15, 177–182. 10.1007/BF03001163 7208128

[B22] Franklin K. B. J. Paxinos G. (2007). The Mouse Brain in Stereotaxic Coordinates, 3rd ed. San Diego, CA: Academic Press.

[B23] Giardino W. J. Eban-Rothschild A. Christoffel D. J. Li S.-B. Malenka R. C. de Lecea L. (2018). Parallel circuits from the bed nuclei of stria terminalis to the lateral hypothalamus drive opposing emotional states. Nat. Neurosci. 21, 1084–1095. 10.1038/s41593-018-0198-x 30038273 PMC6095688

[B24] Girven K. S. Aroni S. Navarrete J. Marino R. A. M. McKeon P. N. Cheer J. F.. (2020). Glutamatergic input from the insula to the ventral bed nucleus of the stria terminalis controls reward-related behavior. Addict. Biol. 26:e12961. 10.1111/adb.12961 32820590 PMC8651178

[B25] Hao S. Yang H. Wang X. He Y. Xu H. Wu X.. (2019). The lateral hypothalamic and BNST GABAergic projections to the anterior ventrolateral periaqueductal gray regulate feeding. Cell Rep. 28, 616.e5–624.e5. 10.1016/j.celrep.2019.06.051 31315042

[B26] Haubensak W. Kunwar P. S. Cai H. Ciocchi S. Wall N. R. Ponnusamy R.. (2010). Genetic dissection of an amygdala microcircuit that gates conditioned fear. Nature 468, 270–276. 10.1038/nature09553 21068836 PMC3597095

[B27] Haufler D. Nagy F. Z. Pare D. (2013). Neuronal correlates of fear conditioning in the bed nucleus of the stria terminalis. Learn. Mem. 20, 633–641. 10.1101/lm.031799.113 24131794 PMC3799415

[B28] Herman J. P. Figueiredo H. Mueller N. K. Ulrich-Lai Y. Ostrander M. M. Choi D. C.. (2003). Central mechanisms of stress integration: hierarchical circuitry controlling hypothalamo-pituitary-adrenocortical responsiveness. Front. Neuroendocrinol. 24, 151–180. 10.1016/j.yfrne.2003.07.001 14596810

[B29] Holland P. C. (1977). Conditioned stimulus as a determinant of the form of the Pavlovian conditioned response. J. Exp. Psychol. Anim. Behav. Process. 3, 77–104. 10.1037//0097-7403.3.1.77 845545

[B30] Jennings J. H. Rizzi G. Stamatakis A. M. Ung R. L. Stuber G. D. (2013a). The inhibitory circuit architecture of the lateral hypothalamus orchestrates feeding. Science 341, 1517–1521. 10.1126/science.1241812 24072922 PMC4131546

[B31] Jennings J. H. Sparta D. R. Stamatakis A. M. Ung R. L. Pleil K. E. Kash T. L.. (2013b). Distinct extended amygdala circuits for divergent motivational states. Nature 496, 224–228. 10.1038/nature12041 23515155 PMC3778934

[B32] Johnson S. B. Emmons E. B. Anderson R. M. Glanz R. M. Romig-Martin S. A. Narayanan N. S.. (2016). A basal forebrain site coordinates the modulation of endocrine and behavioral stress responses via divergent neural pathways. J. Neurosci. 36, 8687–8699. 10.1523/JNEUROSCI.1185-16.2016 27535914 PMC4987438

[B33] Ju G. Swanson L. W. (1989). Studies on the cellular architecture of the bed nuclei of the stria terminalis in the rat: I. Cytoarchitecture. J. Comp. Neurol. 280, 587–602. 10.1002/cne.902800409 2708568

[B34] Kargl D. Kaczanowska J. Ulonska S. Groessl F. Piszczek L. Lazovic J.. (2020). The amygdala instructs insular feedback for affective learning. Elife 9:e60336. 10.7554/eLife.60336 33216712 PMC7679142

[B35] Kessler R. C. Sonnega A. Bromet E. Hughes M. Nelson C. B. (1995). Posttraumatic stress disorder in the National Comorbidity Survey. Arch. Gen. Psychiatry 52, 1048–1060. 10.1001/archpsyc.1995.03950240066012 7492257

[B36] Kim B. Yoon S. Nakajima R. Lee H. J. Lim H. J. Lee Y.. (2018). Dopamine D2 receptor-mediated circuit from the central amygdala to the bed nucleus of the stria terminalis regulates impulsive behavior. Proc. Natl. Acad. Sci. U.S.A. 115, E10730–E10739. 10.1073/pnas.1811664115 30348762 PMC6233075

[B37] Kim J. Lee S. Fang Y.-Y. Shin A. Park S. Hashikawa K.. (2019). Rapid, biphasic CRF neuronal responses encode positive and negative valence. Nat. Neurosci. 22, 576–585. 10.1038/s41593-019-0342-2 30833699 PMC6668342

[B38] Kim J. Pignatelli M. Xu S. Itohara S. Tonegawa S. (2016). Antagonistic negative and positive neurons of the basolateral amygdala. Nat. Neurosci. 19, 1636–1646. 10.1038/nn.4414 27749826 PMC5493320

[B39] Kim S.-Y. Adhikari A. Lee S. Y. Marshel J. H. Kim C. K. Mallory C. S.. (2013). Diverging neural pathways assemble a behavioural state from separable features in anxiety. Nature 496, 219–223. 10.1038/nature12018 23515158 PMC6690364

[B40] Lammel S. Lim B. K. Malenka R. C. (2014). Reward and aversion in a heterogeneous midbrain dopamine system. Neuropharmacology 76(Pt B), 351–359. 10.1016/j.neuropharm.2013.03.019 23578393 PMC3778102

[B41] Lammel S. Lim B. K. Ran C. Huang K. W. Betley M. J. Tye K. M.. (2012). Input-specific control of reward and aversion in the ventral tegmental area. Nature 491, 212–217. 10.1038/nature11527 23064228 PMC3493743

[B42] Lebow M. A. Chen A. (2016). Overshadowed by the amygdala: the bed nucleus of the stria terminalis emerges as key to psychiatric disorders. Mol. Psychiatry 21, 450–463. 10.1038/mp.2016.1 26878891 PMC4804181

[B43] LeDoux J. E. (2000). Emotion circuits in the brain. Annu. Rev. Neurosci. 23, 155–184. 10.1146/annurev.neuro.23.1.155 10845062

[B44] Leibowitz S. F. Hammer N. J. Chang K. (1981). Hypothalamic paraventricular nucleus lesions produce overeating and obesity in the rat. Physiol. Behav. 27, 1031–1040. 10.1016/0031-9384(81)90366-8 7335803

[B45] Namburi P. Al-Hasani R. Calhoon G. G. Bruchas M. R. Tye K. M. (2016). Architectural representation of valence in the limbic system. Neuropsychopharmacology 41, 1697–1715. 10.1038/npp.2015.358 26647973 PMC4869057

[B46] Paton J. J. Belova M. A. Morrison S. E. Salzman C. D. (2006). The primate amygdala represents the positive and negative value of visual stimuli during learning. Nature 439, 865–870. 10.1038/nature04490 16482160 PMC2396495

[B47] Poulos A. M. Ponnusamy R. Dong H.-W. Fanselow M. S. (2010). Compensation in the neural circuitry of fear conditioning awakens learning circuits in the bed nuclei of the stria terminalis. Proc. Natl. Acad. Sci. U.S.A. 107, 14881–14886. 10.1073/pnas.1005754107 20679237 PMC2930410

[B48] Shabel S. J. Janak P. H. (2009). Substantial similarity in amygdala neuronal activity during conditioned appetitive and aversive emotional arousal. Proc. Natl. Acad. Sci. U.S.A. 106, 15031–15036. 10.1073/pnas.0905580106 19706473 PMC2736461

[B49] Shaham Y. Shalev U. Lu L. de Wit H. Stewart J. (2003). The reinstatement model of drug relapse: history, methodology and major findings. Psychopharmacology 168, 3–20. 10.1007/s00213-002-1224-x 12402102

[B50] Tovote P. Esposito M. S. Botta P. Chaudun F. Fadok J. P. Markovic M.. (2016). Midbrain circuits for defensive behaviour. Nature 534, 206–212. 10.1038/nature17996 27279213

[B51] Tovote P. Fadok J. P. Lüthi A. (2015). Neuronal circuits for fear and anxiety. Nat. Rev. Neurosci. 16, 317–331. 10.1038/nrn3945 25991441

[B52] Walker D. L. Davis M. (1997). Double dissociation between the involvement of the bed nucleus of the stria terminalis and the central nucleus of the amygdala in startle increases produced by conditioned versus unconditioned fear. J. Neurosci. 17, 9375–9383. 10.1523/jneurosci.17-23-09375.1997 9364083 PMC6573581

[B53] Williams G. Bing C. Cai X. J. Harrold J. A. King P. J. Liu X. H. (2001). The hypothalamus and the control of energy homeostasis: different circuits, different purposes. Physiol. Behav. 74, 683–701. 10.1016/s0031-9384(01)00612-6 11790431

[B54] Xu Y. Lu Y. Cassidy R. M. Mangieri L. R. Zhu C. Huang X.. (2019). Identification of a neurocircuit underlying regulation of feeding by stress-related emotional responses. Nat. Commun. 10:3446. 10.1038/s41467-019-11399-z 31371721 PMC6671997

[B55] Yuan Y. Wu W. Chen M. Cai F. Fan C. Shen W.. (2019). Reward inhibits paraventricular CRH neurons to relieve stress. Curr. Biol. 29, 1243.e4–1251.e4. 10.1016/j.cub.2019.02.048 30853436

