# Supplementary material for: Dorsal Bed Nucleus of the Stria Terminalis-Subcortical Output Circuits Encode Positive Bias in Pavlovian Fear and Reward
Source: Front Neural Circuits. 2021 Dec 14;15:772512. doi: 10.3389/fncir.2021.772512 (PMC8713515; doi:10.3389/fncir.2021.772512)
Supplement: Supplementary file 1 [file Data_Sheet_1.pdf]

Supplementary Material

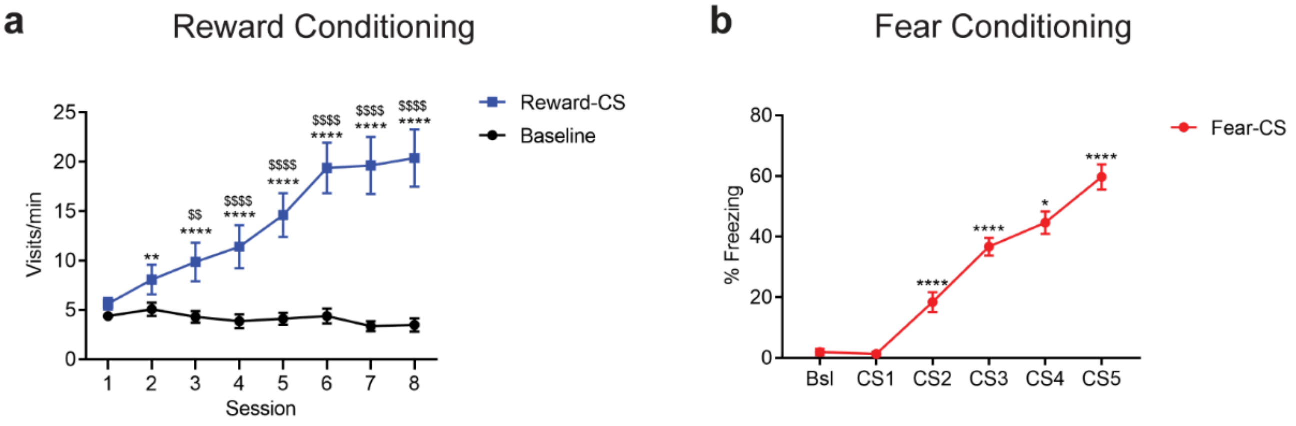

Supplementary Figure 1. Behavioral performance during reward and fear conditioning sessions. (**a**) During reward conditioning, mice in the aCSF and Muscimol groups (n = 24) increased their visit rate per minute across sessions and specifically during reward-CS (two-way RM ANOVA: session, $F_{7,161}$ = 8.077, P < 0.0001; period (baseline vs. Reward-CS), $F_{1,23}$ = 52.36, P < 0.0001; interaction, $F_{7,161}$ = 18.50, P < 0.0001). From session 2, the visit rate during the reward-CS presentation was significantly higher compared to baseline (post hoc two-stage step-up method of Benjamini, Krieger, and Yekutieli ** P < 0.01; **** P < 0.0001) and compared to session 1 ($$ P < 0.01; $$$$ P < 0.0001). (**b**) During fear conditioning, mice increased their freezing levels across the trials (one-way RM ANOVA: trial, $F_{5,115}$ = 88.35, P < 0.0001). From trial 2 (CS2 presentation), freezing levels were consistently higher compared with the previous trial (post hoc two-stage step-up method of Benjamini, Krieger, and Yekutieli * P < 0.05; **** P < 0.0001). Data presented as mean ± SEM.

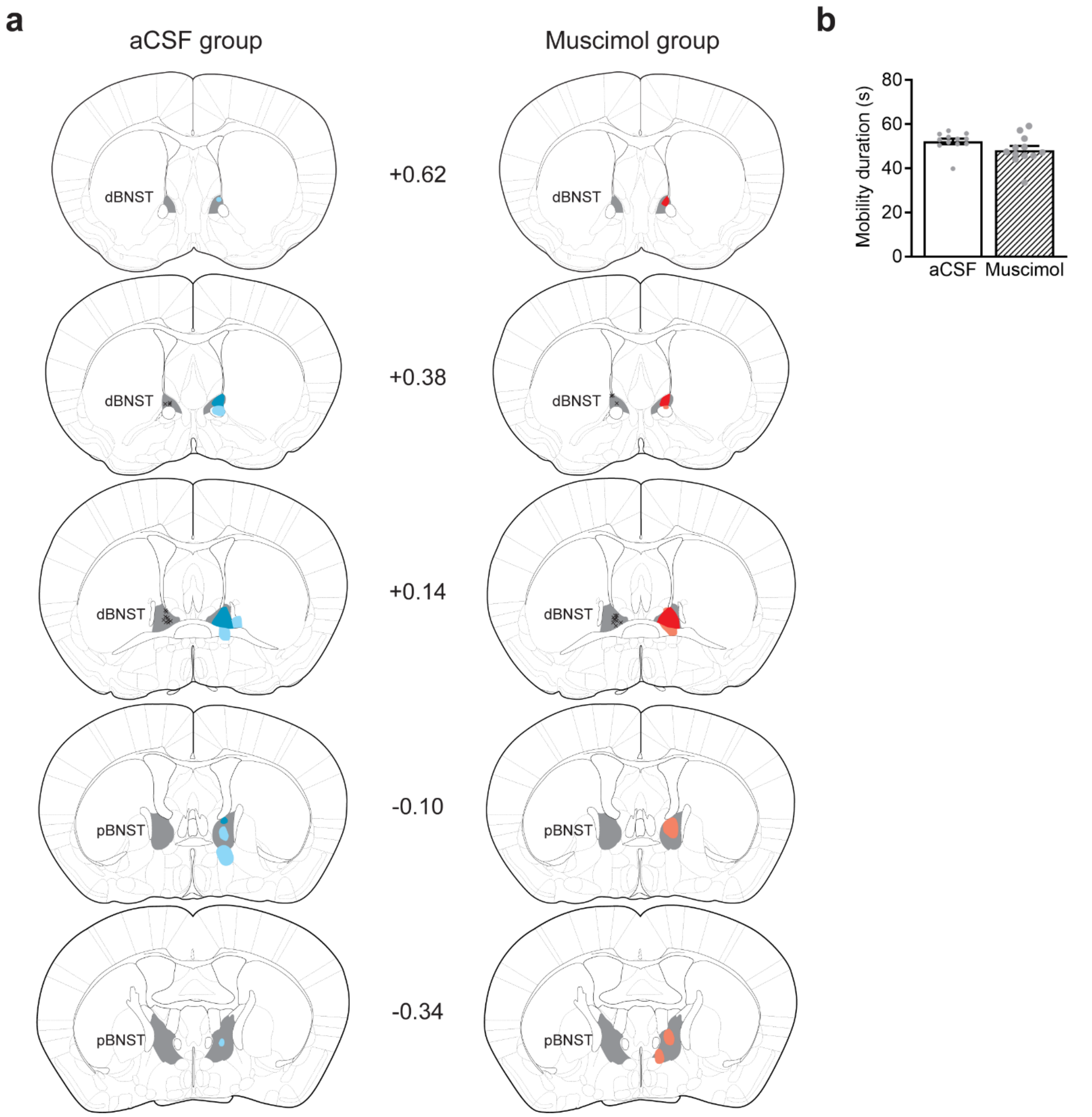

Supplementary Figure 2. Histological and behavioral assessment of intra-dBNST infusions. (a) Histology. On the left side of each brain section, crosses show the center of diffusion area of Muscimol-BODIPY (infused into the dBNST for histological verifications) for each individual mouse. On the right side of those brain sections, blue and red areas indicate the summed diffusion areas of Muscimol-BODIPY observed in all mice in both the aCSF and Muscimol groups. Light blue and orange areas indicate the largest diffusion of Muscimol-BODIPY in the same groups that sometimes occurred in areas adjacent to the dBNST but with lower fluorescent intensity. pBNST: posterior BNST. (**b**) Behavior. Muscimol intra-dBNST infusion did not induce any change in mobility duration during the baseline period of the test (two-tailed unpaired t-test: $t_{22} = 1.716$, $P = 0.1002$).

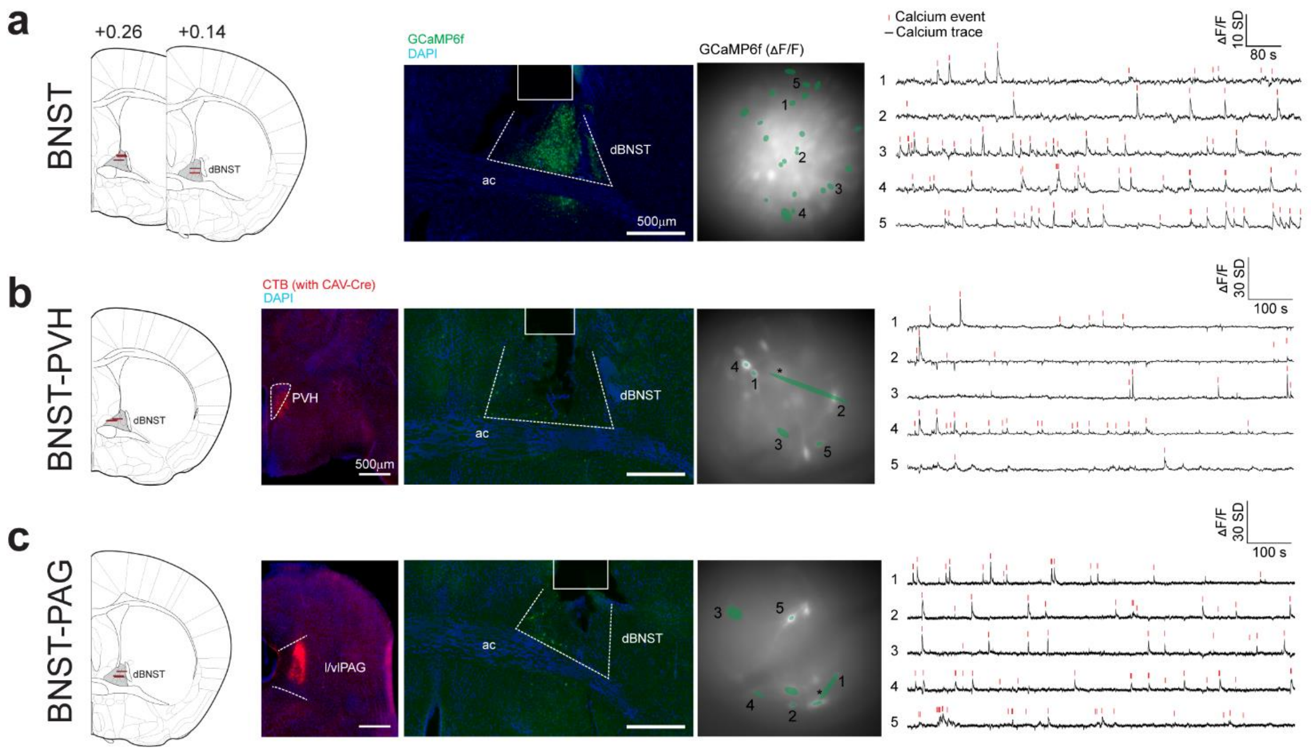

Supplementary Figure 3. Histological assessment of calcium imaging. From left to right: Schematic placement of the microendoscope tip indicated by horizontal red lines for each group ((**a**) BNST, (**b**) BNST-PVH, (**c**) BNST-PAG). CTB-555 expression in PVH (b) or l/vlPAG (c) where it was co-injected with CAV::Cre. GCaMP6f expression with representative lens placement (white rectangle). Dashed lines indicate regional boundaries according to the Allen Brain Atlas. Field of view calcium maximum projection images overlaid with identified units (* indicates putative neurites). Representative calcium traces of five single units for each group. Calcium events are indicated as red bars above peaks.

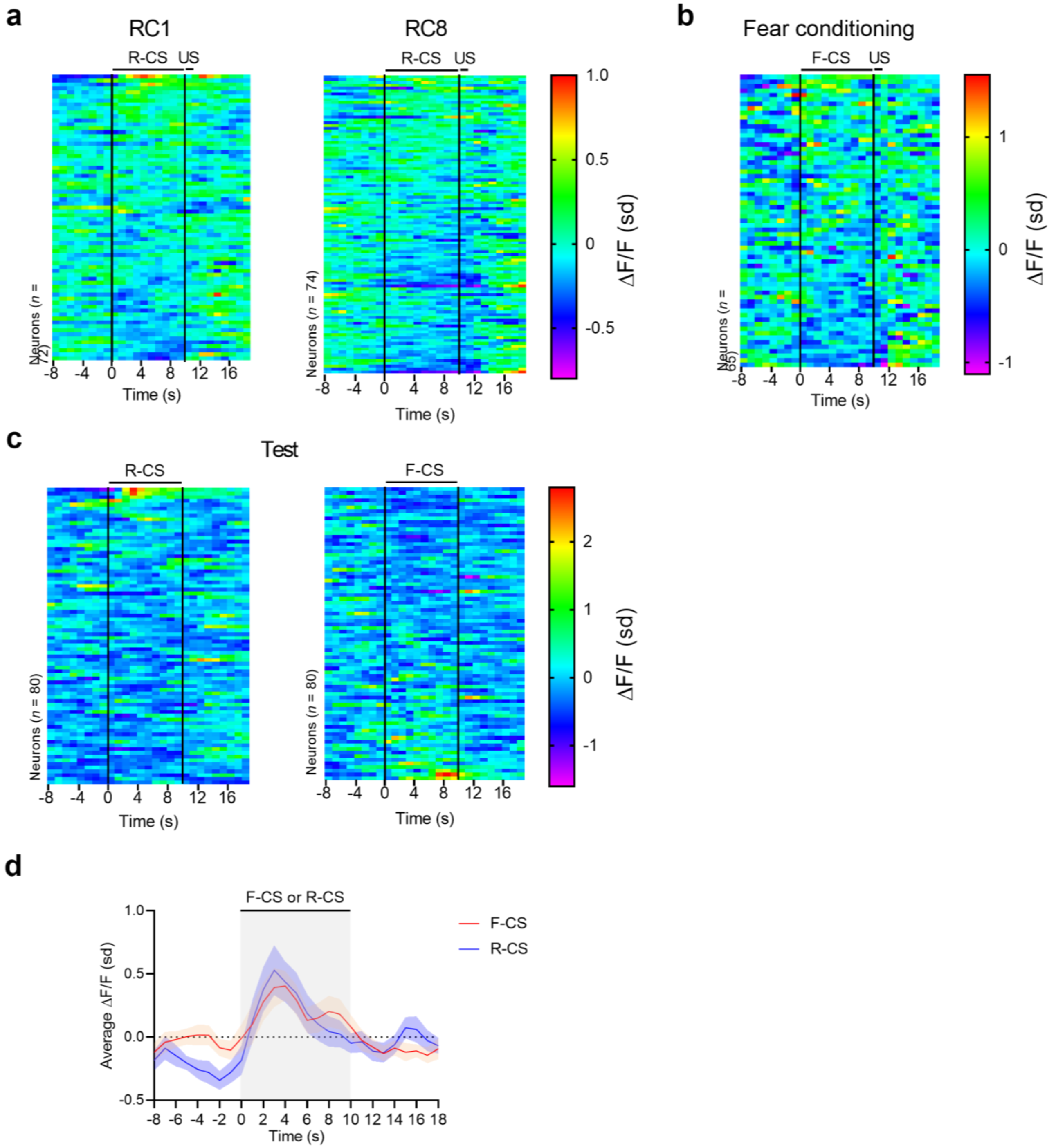

Supplementary Figure 4. Sorted Ca^{2+} traces from dBNST neuronal units from the experiment shown in Figure 2 during reward conditioning (**a**), fear conditioning (**b**) and test (**c**). The units were sorted by their combined CS/US responses (average fear or reward CS/US; a, b) or differences in their CS responses (average reward CS-average fear CS; c). (**d**) Average Ca^{2+} traces (ΔF/F, sd: standard deviation) of CS-responders aligned to F-CS or R-CS onset during the test session.

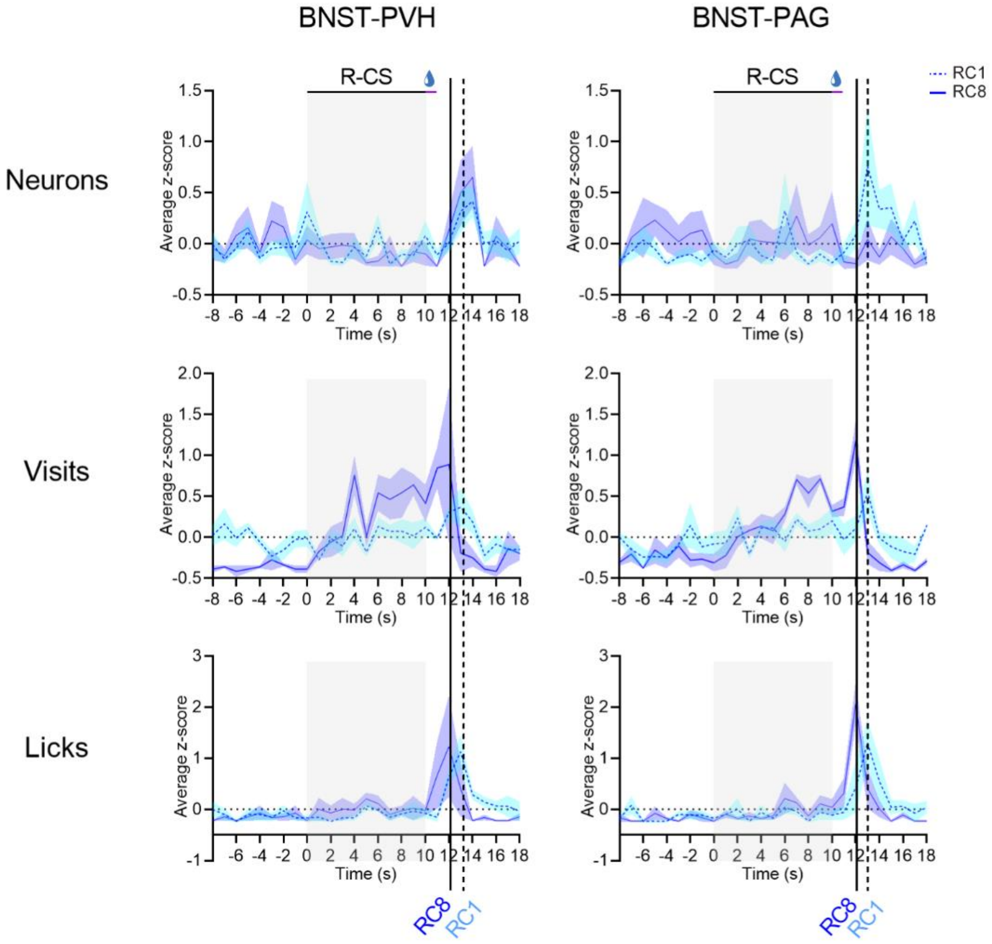

Supplementary Figure 5. Aligning neuronal activity in BNST-PVH and BNST-PAG circuits with behavioral responses. The peak in PETHs of licks (bottom) observed after reward delivery is aligned with the peak in PETHs of visits (middle) and precedes the peak observed in PETHs of population responses (top) during RC1 and RC8 in the BNST-PVH group (left) and during only RC1 in BNST-PAG group (right).

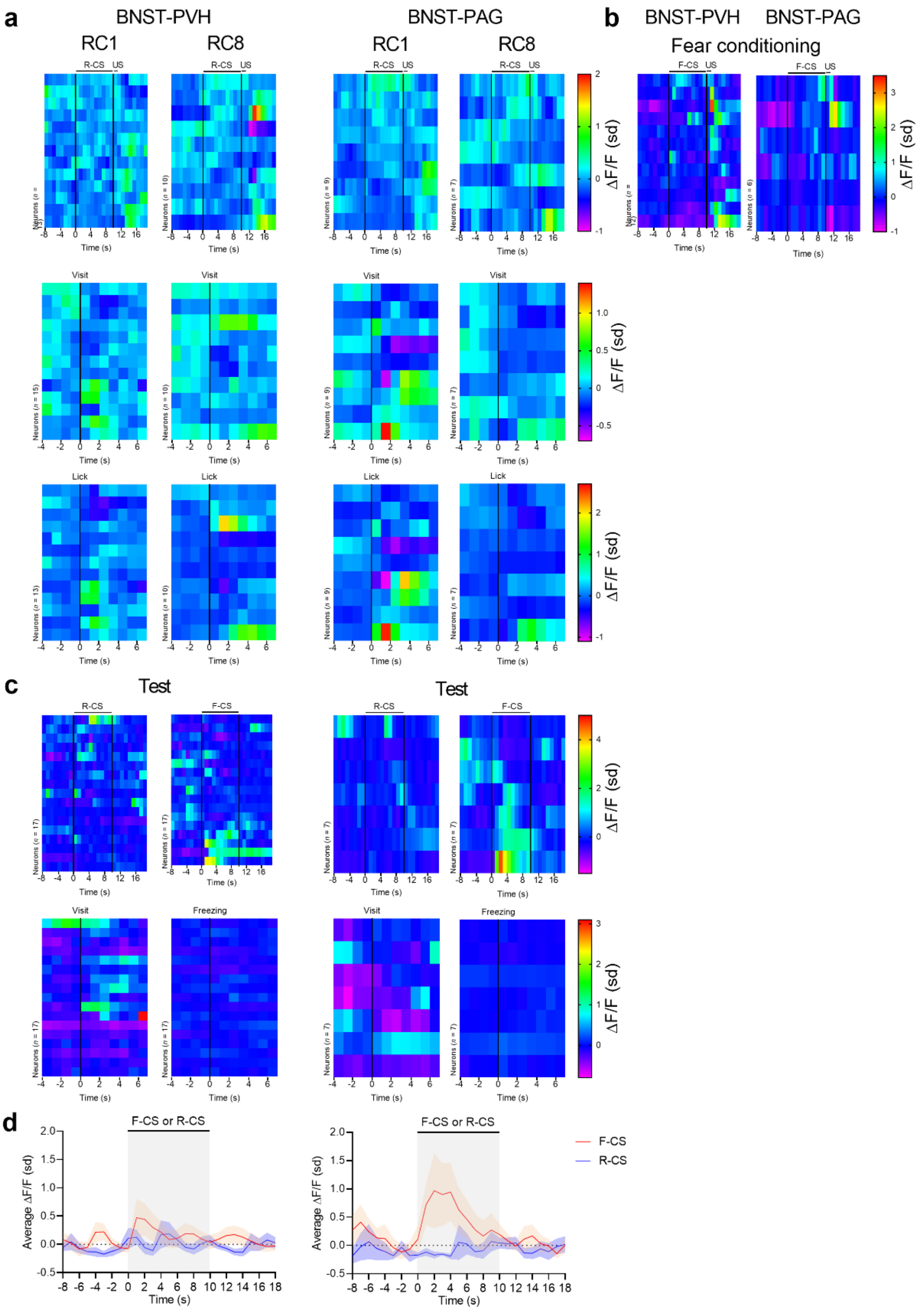

Supplementary Figure 6. Sorted Ca^{2+} traces from BNST-PVH and BNST-PAG neuronal units from the experiment shown in Figure 4 during reward conditioning (**a**), fear conditioning (**b**) and test (**c**). The units were sorted by their combined CS/US responses (average fear or reward CS/US; a, b) or differences in their CS responses (average reward CS-average fear CS; c). (**d**) Average Ca^{2+} traces (ΔF/F, sd: standard deviation) aligned to F-CS or R-CS onset during the test session.

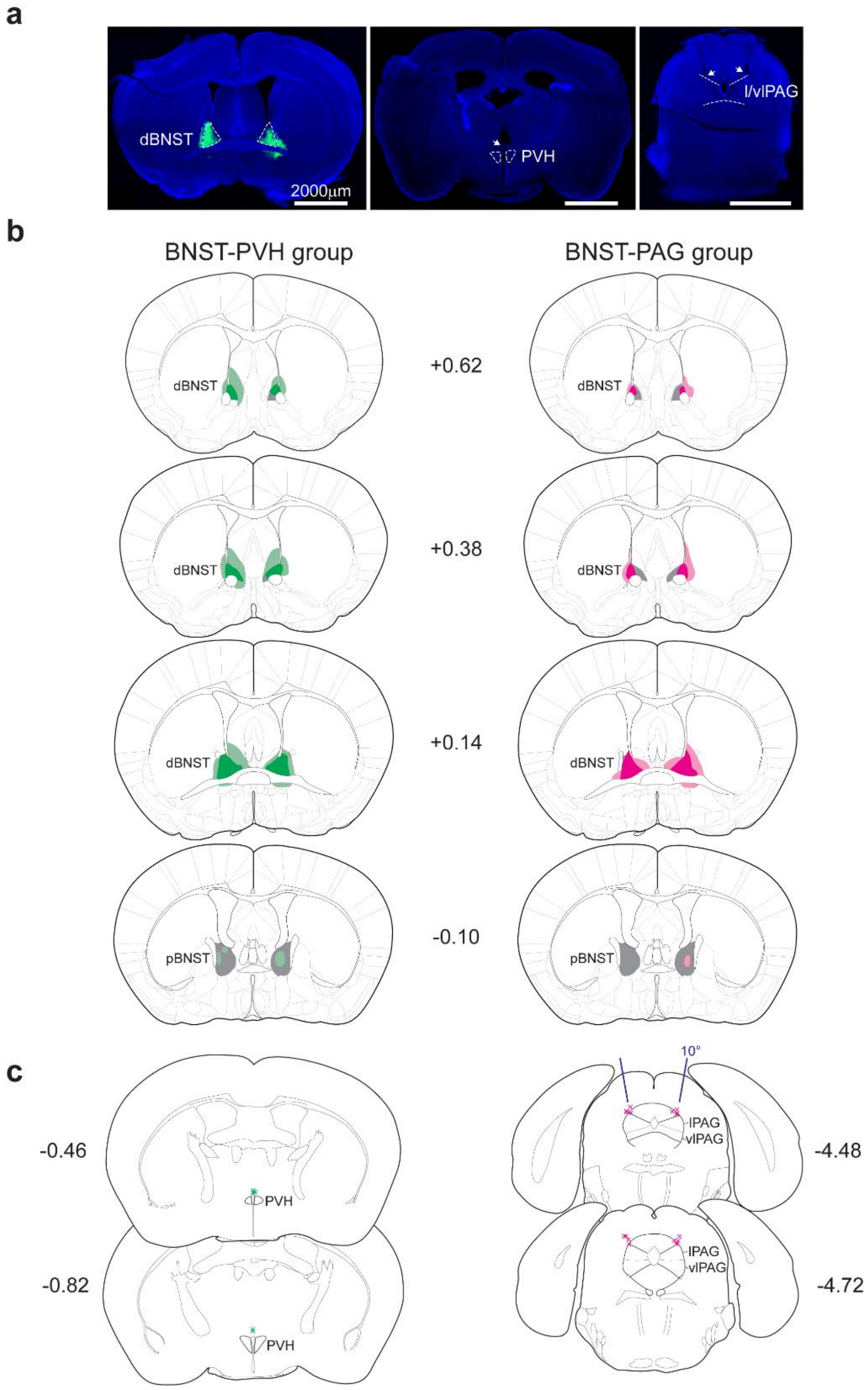

Supplementary Figure 7. Histological assessment of optogenetic manipulations. (**a**) Left, representative pictures showing viral expression in the dBNST. Right, optic fiber tip above the PVH (middle) and bilateral optic fiber tips above the l/vlPAG. (**b**) Group average projections of the minimal (dark green or magenta) and maximal (light green or magenta) extent of ChR2 viral expression from anterior to posterior in BNST-PVH and BNST-PAG groups. (**c**) Schematic of optic fiber tip placements in BNST-PVH group (green crosses) and BNST-PAG group (magenta crosses). The blue lines indicate the 10° angle applied to implantation of the optic fibers above the l/vlPAG.

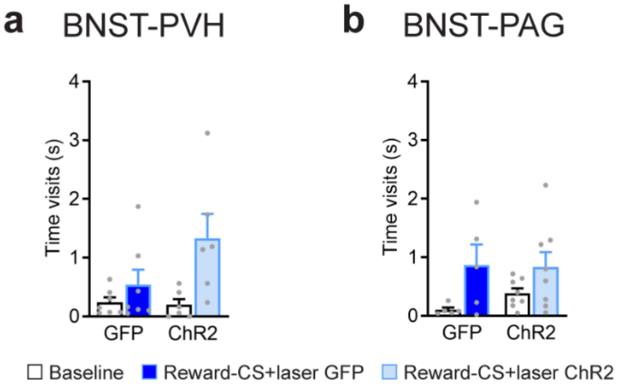

Supplementary Figure 8. Time spent visiting the port during test sessions. All mice increased their time visiting the port compared to the baseline period, (**a**) left, in the BNST-PVH optogenetic experiment (two-way RM ANOVA: period, $F_{1,11} = 8.331$, $P = 0.0148$; group, $F_{1,11} = 2.385$, $P = 0.1508$; interaction, $F_{1,11} = 2.857$, $P = 0.1191$), (**b**) right, and in the BNST-PAG optogenetic experiment (two-way RM ANOVA: period, $F_{1,11} = 7.492$, $P = 0.0193$; group, $F_{1,11} = 0.3080$, $P = 0.59$; interaction, $F_{1,11} = 0.5216$, $P = 0.4852$).

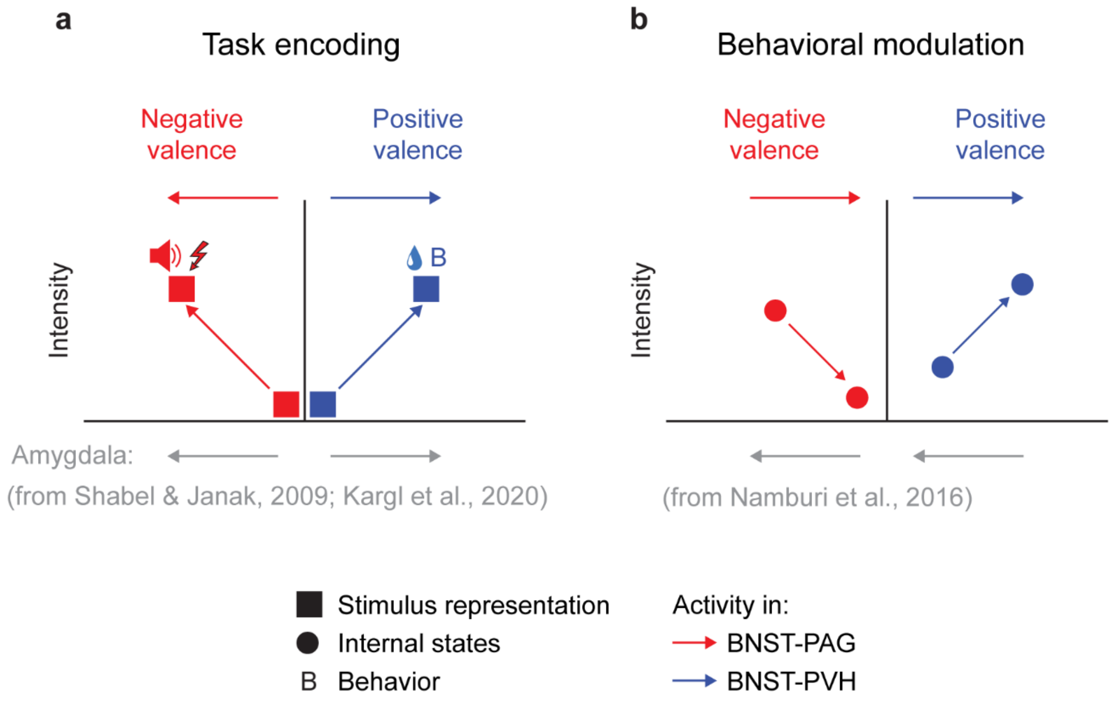

Supplementary Figure 9. Encoding of positive valence bias in dBNST output circuits. Based on our results, we propose that the BNST-PVH circuit encodes reward-related behaviors and the reward US (**a**), drives the execution of reward behaviors (**b**), and associated positive internal states. On the opposite site, the BNST-PAG circuit encodes negatively valenced CSs (**a**), but attenuates negative responding (**b**), and associated negative internal states. Overall, although BNST-PVH and BNST-PAG circuitries differentially encode oppositely valenced stimuli (**a**), they both promote positive bias in Pavlovian responding (**b**). Note that the shock US is encoded by both circuits. In contrast, while differential encoding of positive vs. negative Pavlovian stimuli within the amygdala is similar to what we observed here, its dominant behavioral descending output (to brainstem) is thought to be largely negatively biased (grey).
